# Strongly Confined CsPbBr_3_ Quantum Dots as Quantum Emitters and Building Blocks for Rhombic Superlattices

**DOI:** 10.1021/acsnano.2c07677

**Published:** 2023-01-31

**Authors:** Simon C. Boehme, Maryna I. Bodnarchuk, Max Burian, Federica Bertolotti, Ihor Cherniukh, Caterina Bernasconi, Chenglian Zhu, Rolf Erni, Heinz Amenitsch, Denys Naumenko, Hordii Andrusiv, Nazar Semkiv, Rohit Abraham John, Alan Baldwin, Krzysztof Galkowski, Norberto Masciocchi, Samuel D. Stranks, Gabriele Rainò, Antonietta Guagliardi, Maksym V. Kovalenko

**Affiliations:** † Institute of Inorganic Chemistry, Department of Chemistry and Applied Biosciences, ETH Zürich, 8093 Zürich, Switzerland; ‡ Laboratory for Thin Films and Photovoltaics, Empa, Swiss Federal Laboratories for Materials Science and Technology, 8600 Dübendorf, Switzerland; § Swiss Light Source, Paul Scherrer Institute, 5232 Villigen, Switzerland; ∥ Department of Science and High Technology and To.Sca.Lab., University of Insubria, via Valleggio 11, 22100 Como, Italy; ⊥ Institute of Inorganic Chemistry, Graz University of Technology, 8010 Graz, Austria; # Cavendish Laboratory, University of Cambridge, JJ Thomson Avenue, Cambridge CB3 0HE, U.K.; ∇ Department of Chemical Engineering & Biotechnology, University of Cambridge, Philippa Fawcett Drive, Cambridge CB3 0AS, U.K.; ⊗ Istituto di Cristallografia and To.Sca.Lab, Consiglio Nazionale delle Ricerche, via Valleggio 11, 22100 Como, Italy; †† Electron Microscopy Center, Empa, Swiss Federal Laboratories for Materials Science and Technology, 8600 Dübendorf, Switzerland

**Keywords:** quantum confinement, perovskites, colloidal nanocrystals, excitons, self-assembly

## Abstract

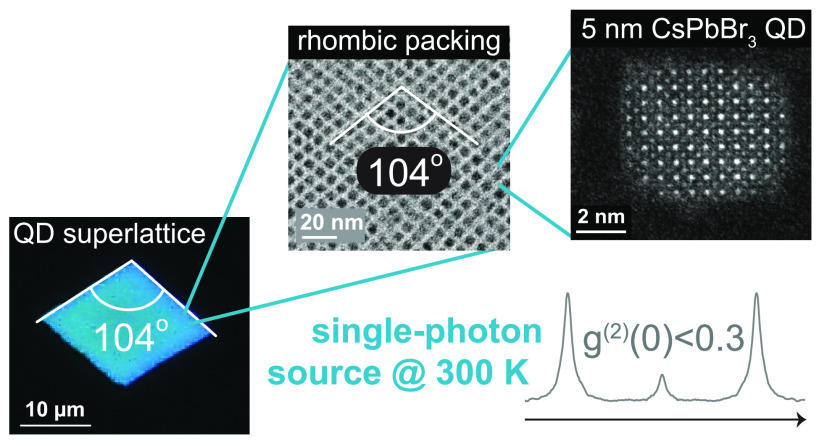

The success of the colloidal semiconductor quantum dots (QDs) field is rooted in the precise synthetic control of QD size, shape, and composition, enabling electronically well-defined functional nanomaterials that foster fundamental science and motivate diverse fields of applications. While the exploitation of the strong confinement regime has been driving commercial and scientific interest in InP or CdSe QDs, such a regime has still not been thoroughly explored and exploited for lead-halide perovskite QDs, mainly due to a so far insufficient chemical stability and size monodispersity of perovskite QDs smaller than about 7 nm. Here, we demonstrate chemically stable strongly confined 5 nm CsPbBr_3_ colloidal QDs via a postsynthetic treatment employing didodecyldimethylammonium bromide ligands. The achieved high size monodispersity (7.5% ± 2.0%) and shape-uniformity enables the self-assembly of QD superlattices with exceptional long-range order, uniform thickness, an unusual rhombic packing with an obtuse angle of 104°, and narrow-band cyan emission. The enhanced chemical stability indicates the promise of strongly confined perovskite QDs for solution-processed single-photon sources, with single QDs showcasing a high single-photon purity of 73% and minimal blinking (78% “on” fraction), both at room temperature.

Since 2015, intense research on colloidal lead-halide perovskite (LHP) nanocrystal (NC) quantum dots (QDs)^1^ has enabled significant progress in their synthesis, fundamental understanding of their electronic structure,^2−4^ and a variety of proof-of-principle applications.^5−8^ However, while a plethora of works on LHP QDs has focused on the weak-to-intermediate confinement regime, with QD sizes equal to or larger than the Bohr diameter (∼7 nm in CsPbBr_3_),^1^ only a few reports have explored LHP QDs in the strong confinement regime,^9,10^*i.e.*, QDs of about 5 nm and below. For example, while larger QDs have already displayed outstanding properties such as long coherence times,^11,12^ bright single-photon emission,^13,14^ self-assembly into QD superlattices (SLs),^15−17^ collective emission via superfluorescence,^18,4,17^ and superior performance in displays and LEDs,^19−23^ our understanding of the performance of strongly confined QDs is lagging behind, in each of these categories. Experimentally revealing the structural and optical properties of strongly confined QDs could consolidate current theoretical models on the energy-level ordering of fine-structure split (FSS) band-edge excitons,^2^ as well as clarify the strength of exciton–phonon coupling^24−28^ and Coulomb many-body interactions.^29,30^ Both of the latter are crucial for the guided design of photonic devices operating in the weak light–matter coupling regime, *e.g.*, to improve the performance of cyan-emitting LEDs, and/or in the strong coupling regime, *e.g.*, to demonstrate single-photon nonlinearities.^31^

Such a lack of knowledge regarding expressions and use of strong excitonic confinement in LHP QDs relates to the presently limited synthetic access to the strong confinement regime. Challenges arise from both the rapid growth kinetics and the structural instability of small LHP QDs with a large surface-to-volume ratio, especially for <7 nm QDs passivated with conventional ligands, *i.e.*, oleic acid and oleylamine. While the rapid growth kinetics has recently been addressed by introducing a synthesis based on thermodynamic, rather than kinetic, control,^9^ the structural instability remains an unresolved challenge limiting both structural studies (*e.g.*, by electron microscopy and X-ray diffraction) and optical investigations (*e.g.*, by single-emitter PL spectroscopy). Furthermore, reports on small QDs are still plagued by an insufficient monodispersity in size and shape, largely preventing their self-assembly into superlattices.

In this study, we focus on 5 nm CsPbBr_3_ QDs and stabilize them by the postsynthetic surface treatment with didodecyldimethylammonium bromide (DDAB). The resulting QDs are slightly oblate in shape and exhibit well-resolved absorption features, narrowband cyan PL, good long-term colloidal and optical stability, and a high PL quantum yield (PLQY). Owing to high size monodispersity (7.5% ± 2.0%) and shape-uniformity, these QDs readily form SLs exhibiting exceptional long-range order and narrowband PL (full-width-at-half-maximum (fwhm) ∼100 meV, quasi-identical with the fwhm of single QDs). Unconventionally, the SLs exhibit a macroscopic oblique shape with a characteristic obtuse angle of about 104°, resulting from an unexpected QD packing, consistent with a C-centered rectangular lattice in the (transmission electron microscopy; TEM) observation plane. For reference, larger CsPbBr_3_ NCs typically form simple cubic SLs.^15−17^

*Via* single-QD PL spectroscopy, we show that strongly confined colloidal 5 nm CsPbBr_3_ QDs can serve as solution-processed single-photon sources operating at room temperature. While recent theoretical efforts predicted a crossover from a bright triplet to a dark singlet ground state for small QDs,^2,32^ our 5 nm QDs still exhibit short radiative lifetimes of ∼0.5 ns at cryogenic temperature. Hence, the dark state marginally contributes to the radiative recombination at zero magnetic field even for strongly confined CsPbBr_3_ QDs, possibly linked to a reverse dark-bright energy-level ordering^2,33^ or to a forbidden bright-to-dark relaxation.^34^

## Results and Discussion

### Postsynthetic Treatment, QD Size, Shape, and Crystal Structure

Motivated by earlier works demonstrating high PLQY and good long-term colloidal stability of CsPbX_3_ NCs with a didodecyldimethylammonium bromide (DDAB) ligand shell,^35^ we posited that DDAB ligands may render also the small CsPbBr_3_ QDs more robust while preserving high emissivity. Briefly, 5 nm CsPbBr_3_ QDs were synthesized using a hot-injection approach by adapting the methodology from Dong et al.,^9^ using conventional oleylammonium-based ligand capping. Subsequent surface treatment with DDAB in toluene utilizes the stronger affinity of the DDA-cation to the surface A-site.^36^

**Figure 1:**
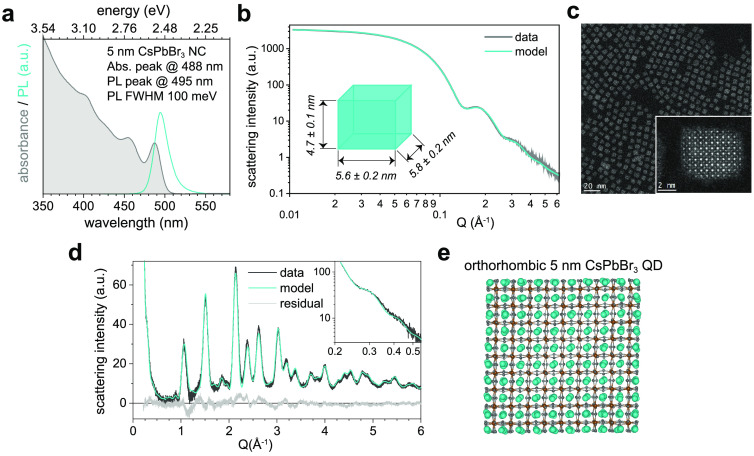
Strongly confined DDAB-capped 5 nm CsPbBr_3_QDs with narrow-band PL and well-resolved absorption features. (a) Absorbance (gray) and PL (cyan) spectra. (b) Fitting the experimental SAXS data of a QD dispersion (gray line) via an analytical model (cyan line) allows for particle shape determination (see cyan particle model in the inset), yielding an oblate shape with edge lengths of 4.7 ± 0.1 nm, 5.6 ± 0.2 nm, and 5.8 ± 0.2 nm, respectively. (c) High-resolution STEM image, with a single QD shown in the inset. (d) The Porod small-angle region and the WAXTS experimental data (black line) simultaneously fitted via the DSE (cyan line, residuals: gray line), utilizing atomistic models of QDs^37^ with an orthorhombic crystal structure (see main text and Supporting Information for details). (e) Atomistic model of a size-averaged QD, obtained from the fit in (d) and a subsequent geometry relaxation at the DFT/PBE level of theory; Cs, Pb, and Br atoms are depicted in cyan, brown, and gray, respectively.

Figure 1a exemplifies strong confinement of excitons in 5 nm CsPbBr_3_ QDs, *i.e.*, QDs smaller than the Bohr diameter (∼7 nm) in this material.^1^ First, a large bandgap is observed in absorption (with a lowest-energy peak at 488 nm) and PL (with a peak at 495 nm), manifested as cyan-blue appearance of a colloidal dispersion under daylight and UV light; second, strongly confined 5 nm QDs exhibit clearly discernible absorption features, due to the sparse density of states in these QDs. At the ensemble level, such a discretized absorption spectrum as well as the small PL fwhm of about 100 meV can only be observed in the case of a sufficiently low size polydispersity. From experimental small-angle X-ray scattering (SAXS) data of a QD dispersion in toluene and the associated fit via an analytical model (see Figure 1b), we derive an oblate QD shape (parallelepiped) with edge lengths of 4.7 ± 0.1 nm, 5.6 ± 0.2 nm, and 5.8 ± 0.2 nm, respectively (1:1.19:1.23 ratio), and a low size dispersity of 7.5% ± 2.0% of the ensemble. For simplicity, this shape with an equivalent length of the two long axes can be then viewed as a flat tetragonal prism of *ca*. 1:1.2 = 0.8 aspect ratio. The particle size and shape are consistent with high-resolution scanning transmission electron microscopy (HRSTEM), see Figure 1c, and wide-angle X-ray total scattering (WAXTS) experiments, see Figure 1d. Fitting the Porod small-angle region of the WAXTS experimental data via the Debye scattering equation (DSE) and utilizing atomistic models of defective QDs^37^ exposing six cubic facets^38^ yields an oblate shape, with derived QD size and size distributions identical, within experimental error, to the SAXS-derived values in Figure 1b. The best WAXTS-DSE fit provides the atomistic model of a size-averaged QD, which, after geometry relaxation at the density functional theory (DFT/PBE) level of theory, is shown in Figure 1e.

### QD Superlattices

**Figure 2:**
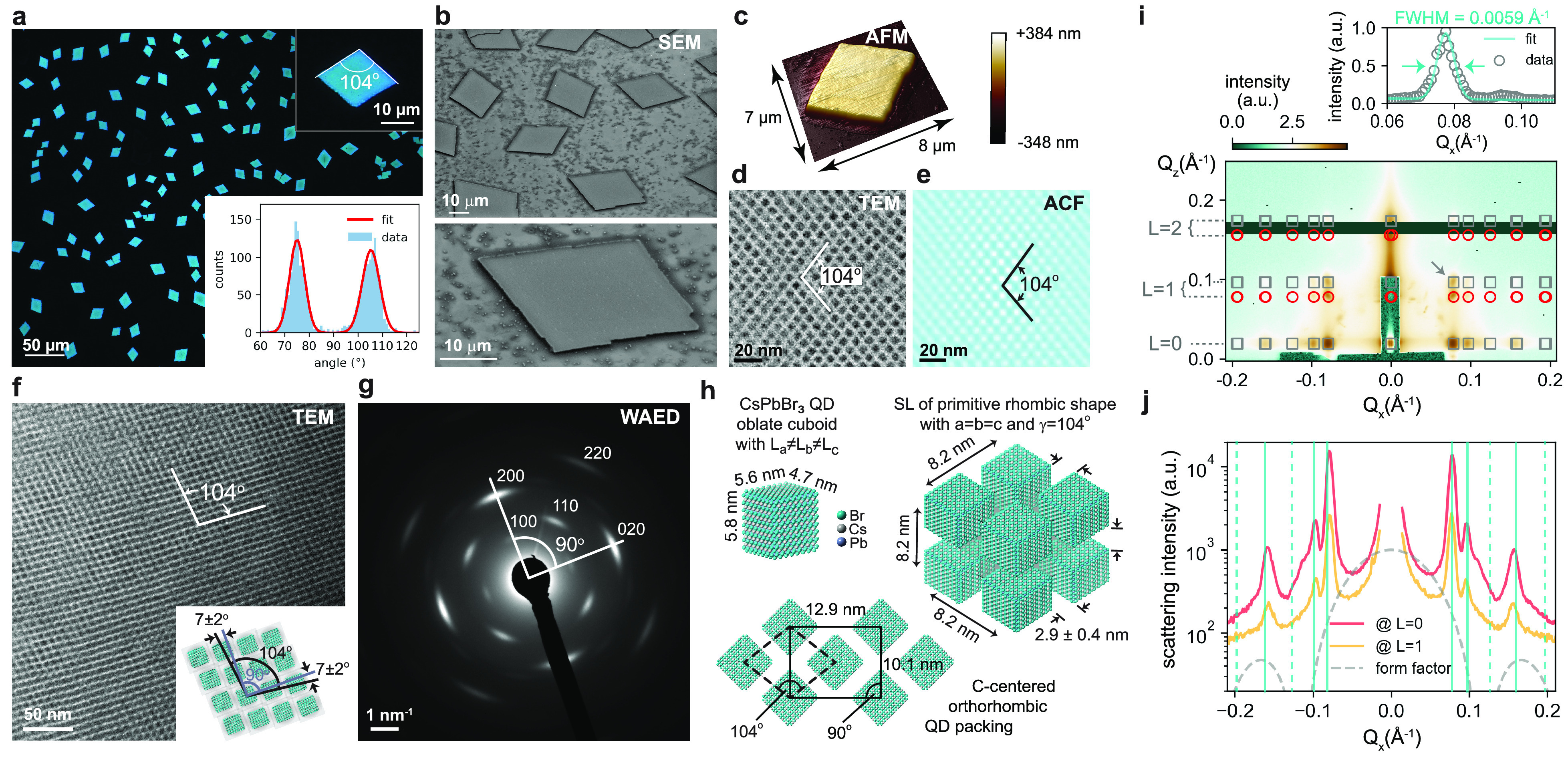
Self-assembly of rhombic QD SLs from cuboidal 5 nm CsPbBr_3_ QDs. (a) Optical microscopy image of 3D QD SLs under UV excitation; the upper inset shows a magnified view of a representative SL to better visualize the nonorthogonal in-plane angle of about 104°; the lower inset shows an angle histogram of all SLs in the frame. (b) SEM images and (c) AFM image of representative SLs. (d) TEM image of a 2D QD SL and (e) its associated autocorrelation function (ACF), both featuring QD packing with an in-plane angle of about 104°. (f) Larger-area TEM image; *inset*: suggested structural model based on an overlay of the axes of QD lattice (gray) and SL (black), inferred from the images in (f) and (g), respectively, with a relative tilt of about 7 ± 2°. (g) WAED at the same sample position and orientation as in (f) suggests that the atomic order of bulk CsPbBr_3_ is largely preserved, as demonstrated by the (pseudo)cubic indexing of the WAED reflections. (h) Suggested structural model of the rhombic QD SL, based on the size and shape of the oblate QDs (from SAXS, Figure 1b) and the QD packing (from TEM and WAED); the average inter-QD facet-to-facet separation of 2.9 ± 0.4 nm is equal in all three spatial directions (*a* = *b* = *c* = 8.2 nm); the QD packing with rhombic repeating units (black dashed rhombus) exhibits a C-centered orthorhombic symmetry (black solid rectangle). (i) 2D GISAXS pattern (with false colors representing the diffracted intensity) demonstrating high long-range in-plane and out-of-plane order; gray and red markers indicate diffraction patterns resulting from reflected and transmitted channel, respectively; *upper panel*: a Gaussian fit to the orthorhombic 201_SL_ peak (*L* = 1; indicated with an arrow in the lower panel) yields a fwhm of 0.0059 Å^–1^ corresponding to a lower limit for the coherent SL domain size of *ca*. 106 nm. (j) 1D in-plane scattering intensity obtained from horizontal cuts at *L* = 0 (solid red line) and *L* = 1 diffraction order (transmitted light only, solid orange line). Comparison with the in-plane cuboidal QD shape form factor (gray dashed line) explains the intensity variation of both pronounced (indicated by solid vertical lines) and suppressed peaks (dashed vertical lines), particularly at about ±0.12 Å^–1^.

A high size and shape uniformity of QDs is also a key prerequisite for a controlled nucleation and growth of high-quality QD SLs. Indeed, the postsynthetic DDAB treatment facilitates the self-assembly of 5 nm CsPbBr_3_ QDs into three-dimensional SLs with exceptional long-range order. These SLs exhibit an unconventional macroscopic oblique shape with a characteristic obtuse angle of ∼104° (see optical microscopy images in Figure 2a), a lateral size of up to 30 μm (see scanning electron microscopy (SEM) images in Figure 2b), and a thickness of several hundreds of nanometers with a root-mean-square roughness of about 10 nm (see atomic force microscopy (AFM) images in Figure 2c and Figure S1). The observation of an oblique SL shape is atypical considering that larger QDs tend to assemble into ordered three-dimensional cuboidal structures with simple cubic packing.^4,18,39^ Nevertheless, we note that the overall symmetry lowering (compared to a primitive cubic packing) is only moderate, given that the overall SL periodicity is consistent with a C-centered cell (space group *Cmmm*), after a simple SL cell transformation from the oblique **a**, **b**, **c** axes to the orthorhombic axes: **a**_**0**_ = **a** + **b**, **b**_**0**_ = **a** – **b**, and **c**_**0**_ = **c**, respectively, all perpendicular to each other (*vide infra*).

To investigate the microscopic nature and origin of the atypical SL assembly, we performed TEM, wide-angle electron diffraction (WAED), and grazing-incidence small-angle X-ray scattering (GISAXS) experiments. TEM on a monolayer-thick “2D SL” (see Figures 2d and 2e) reveals that QDs pack along in-plane axes with an enclosed obtuse angle of 104°. The in-plane packing in 3D SLs is likely identical, since the obtuse angle of the in-plane packing in 2D SLs (Figure 2d) matches that of the macroscopic shape of 3D SLs (see Figure 2a). WAED (see Figure 2g) collected from a representative 2D SL region (see Figure 2f) infers orthogonal angles, consistent with the orthorhombic crystal structure, (see Figure 1d). To simplify the analysis in Figure 2g, we utilize a cubic notation^38^ to assign *hkl* indices to ED reflections; such an approximation is justified given that the small difference between the orthorhombic (010)_ortho_ and (101)_ortho_*d*-spacings is hardly detectable by WAED for small QDs. The intense spots and only short arc-like elongations of the 110_cubic_, 020_cubic_, and 220_cubic_ reflections attest a high degree of orientational order of the QDs within the SL. An overlay of atomic packing and QD packing (see inset in Figure 2f) explains the 104° SL angle via a slight misorientation of the in-plane QD and SL axes, on average by about 7 ± 2° per axis. GISAXS measurements (see Figures 2i and 2j) reveal an exceptional long-range order, both in plane and out of plane. The 2D GISAXS pattern in Figure 2i suggests lattice constants of equal length (*a* = *b* = *c* = 8.2 nm), an in-plane angle of 104°, orthogonal out-of-plane angles, and a lower limit for the coherent SL domain size of ∼106 nm.

Based on the TEM, WAED, and GISAXS evidence, Figure 2h depicts the SL 3D structural model. Due to the partial misorientation of the oblate QDs within the SL, the inter-QD separations vary, with average facet-to-facet separations of about 2.9 ± 0.4 nm, *i.e.*, about 1.7 times the length of a free DDAB molecule (1.71 nm). However, the minimum separation of “diagonally seated” QDs in the [110]_SL_ direction reduces to 2.5 ± 0.3 nm, *i.e.*, only 1.5 times the DDAB ligand length. Such a separation significantly shorter than twice the ligand length may originate from ligand tails bending away from the axis of QD contact.^40,41^

As reported previously for metallic, metal-oxide, and metal-chalcogenide QD SLs, a rhombic packing of cuboidal QDs increases the packing density,^42−45^ possibly favored by the flexible ligand shell. To appreciate the importance of the latter in small-QD SLs, we define the QD softness as λ = 2*L*_lig_/*l*_QD_, with *L*_lig_ the capping ligand length and *l*_QD_ the QD edge length.^17^ Compared to ∼9 nm QDs (λ ∼ 0.4), known to pack in (primitive) cubic assemblies,^17^ the SLs of 5 nm QDs (λ ∼ 0.65) are significantly softer, hereby facilitating also dense noncubic assemblies. Noteworthy, unusually high packing densities even in large-QD SLs have recently been attributed to deformations and vortices of the ligand shell as introduced in the orbifold topological model.^40,41^ Extending such theoretical efforts also to soft small-QD SLs as reported here, ideally with atomistic detail, could be an interesting avenue toward predicting, designing, and realizing hitherto unexplored SL structures.

### 5 nm CsPbBr_3_ QDs at the Single-Particle/Single-Photon Level

Unlike state-of-the-art single-photon sources based on III–V semiconductor QDs, CsPbBr_3_ QDs may emit single photons not only at cryogenic,^11,14^ but also at room temperature,^13,46,47^ a key advantage toward a more widespread deployment in quantum technologies. Entering the strong quantum-confinement regime could unlock additional benefits, since enhanced Auger recombination of multiexcitons for smaller QDs should enhance their single-photon purity. However, only a few reports have explored strongly confined perovskite NCs at the single-particle level, focusing either only on single-photon purity of QDs at room temperature,^48,49^ or on the exciton fine structure of nanoplatelets at cryogenic temperatures.^50^

**Figure 3:**
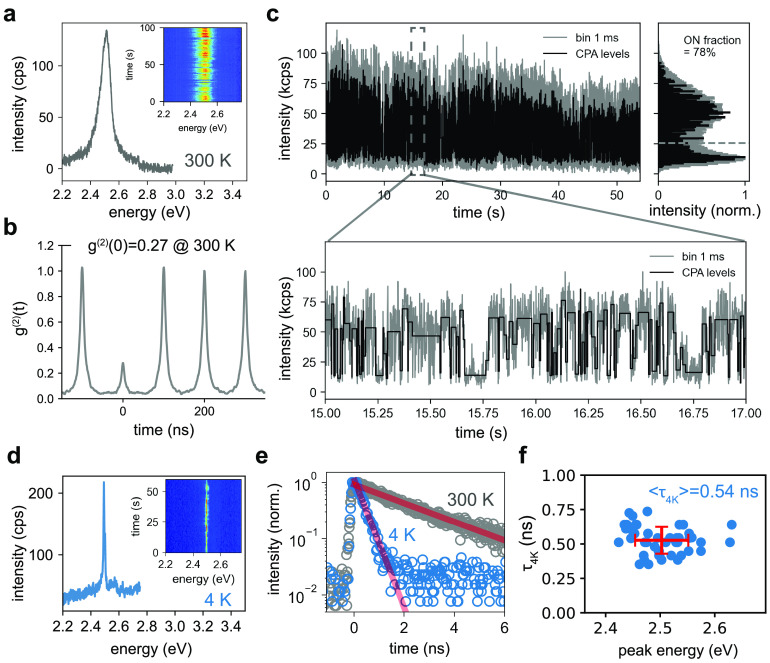
Strongly confined single 5 nm QDs as single-photon sources. (a) Room-temperature PL spectrum of a single QD after pulsed excitation (405 nm, 10 MHz); *inset:* time trace over 100 s, showing spectrally stable emission and several blinking events. (b) Second-order correlation function *g*^(2)^(*t*) with *g*^(2)^(0) = 0.27, indicative of the emission of single photons from single QDs at room temperature. (c) *Upper left panel:* intensity–time trace showing the blinking of a single QD at room temperature, obtained via either 1 ms time binning (gray trace) or via a bias-free Bayesian change-point analysis algorithm (CPA, black trace) adapted from Palstra et al.;^55^*upper right panel:* a 78% ON state fraction is derived from the count histogram with a threshold indicated by the gray dashed line; *lower panel*: a magnified view of the time span indicated by the gray dashed box in the upper panel. (d) PL spectrum of a single QD at 4 K; *inset*: time trace over 60 s. (e) Time-resolved PL of a single QD at 4 K (blue circles) and 300 K (gray circles), respectively; the initial decays are well fitted by single-exponential decays (red lines) with time constants of ∼0.4 ns and ∼2.6 ns, respectively. (f) Statistics of the PL lifetime and peak energy of several single QDs at 4 K (blue circles), with the mean and standard deviation shown in red.

Here, we report single-QD PL properties both at room and cryogenic temperatures (Figure 3). Figure 3a presents the room-temperature PL spectrum of a single 5 nm CsPbBr_3_ QD with a representative fwhm of ∼85 meV, reducing to about 2 meV at 4 K, see Figure S14.^51,52^ Afforded by DDAB surface passivation and encapsulation in a N_2_ environment (see Methods section for details), the QD displays little spectral diffusion and no blue-shifts, *i.e.*, irreversible photodegradation via a progressive decrease in QD core size (see inset). Figure 3b shows the second-order PL intensity correlation function *g*^(2)^(*t*) obtained in a Hanbury Brown and Twiss setup under pulsed excitation at 405 nm with a repetition rate of 10 MHz. The antibunching peak with *g*^(2)^(0) = 0.27 corresponds to a single-photon purity of 73%, a clear improvement compared to the weak confinement regime.^49^

As shown in Figure 3c, the PL intensity intermittency, *i.e.*, “blinking”, in a single 5 nm CsPbBr_3_ QD displays a high ON/OFF ratio, qualitatively similar to larger CsPbBr_3_ QDs.^53,54^ In the first method to derive this ratio, we choose a fix time binning (1 ms, see gray traces in Figure 3c), short enough to ensure a reasonably low probability of capturing both ON and OFF state in a single time bin; such a method is commonly employed in single-emitter spectroscopy. The second method leverages current developments toward bias-free and model-free statistical analysis of QD blinking, for which we adapt a recently introduced algorithm by Palstra et al.,^55^ implementing Bayesian changepoint analysis (CPA) and level clustering. The histogram in Figure 3c illustrates that both analysis schemes reveal a distribution of ON and OFF states and, importantly, a high ON fraction of about 70–80%. Cooling to 4 K does not significantly increase the ON fraction further, see Figure S15.

Next, we study cuboidal 5 nm CsPbBr_3_ QDs also at 4 K (see Figures 3d–f). At such cryogenic temperatures, the recently uncovered fast emission from bright triplet exciton states in CsPbBr_3_ QDs^33^ has sparked efforts to exploit LHP QDs as bright and coherent quantum-light sources.^11^ The unusually fast radiative decay initiated a lively debate on the dark-bright energy-level ordering of LHP QDs in the weak-to-intermediate confinement regime.^33,34,56^ Adding to the complexity, recent theoretical work has suggested that the fine-structure splitting and the dark-bright energy-level order is size-dependent, with strongly confined CsPbBr_3_ QDs exhibiting a dark singlet ground state several millielectronvolts below the bright triplet state.^2^ If true, a first-order estimate for our 5 nm QDs would predict a slow radiative decay from the singlet state, with a PL lifetime significantly exceeding the few hundreds of picoseconds of weakly confined LHP QDs^11^ and similar to the case of metal chalcogenide^57−59^ and InP^60^ QDs. In contrast to such expected size-dependent trends, 5 nm QDs still possess a short PL lifetime of ∼0.5 ± 0.1 ns (see Figure 3e and 3f). Hence, if the singlet state would indeed be below the triplet state, the triplet-to-singlet transition must be exceedingly slow,^34,61^*i.e.*, slower than the ∼0.5 ns radiative decay. Recently, the latter scenario has been invoked to explain the absence of low-energy dark emission in LHP QDs at zero magnetic field and attributed to a strong phonon bottleneck.^34,61^ While such a model is, in principle, consistent with our data, the origin of a phonon bottleneck merits further studies given that the expected dark-bright splitting in strongly confined QDs is of a similar magnitude (few millielectronvolts) as the available low-energy optical phonons.^34,52,62,63^ In either case, the sub-ns lifetime and high PLQY suggest that even strongly confined CsPbBr_3_ QDs may belong to the family of bright emitters with high radiative rates.

Upon heating to room temperature, the PL decay decelerates only slightly to ∼2.6 ns (1/e time), which, together with the high PLQY, places an upper limit to the radiative decay of ∼5 ns, *i.e.*, suggests faster room-temperature emission than for larger CsPbBr_3_ QDs.^4^

### Uniform Optical Properties of QD Superlattices

**Figure 4:**
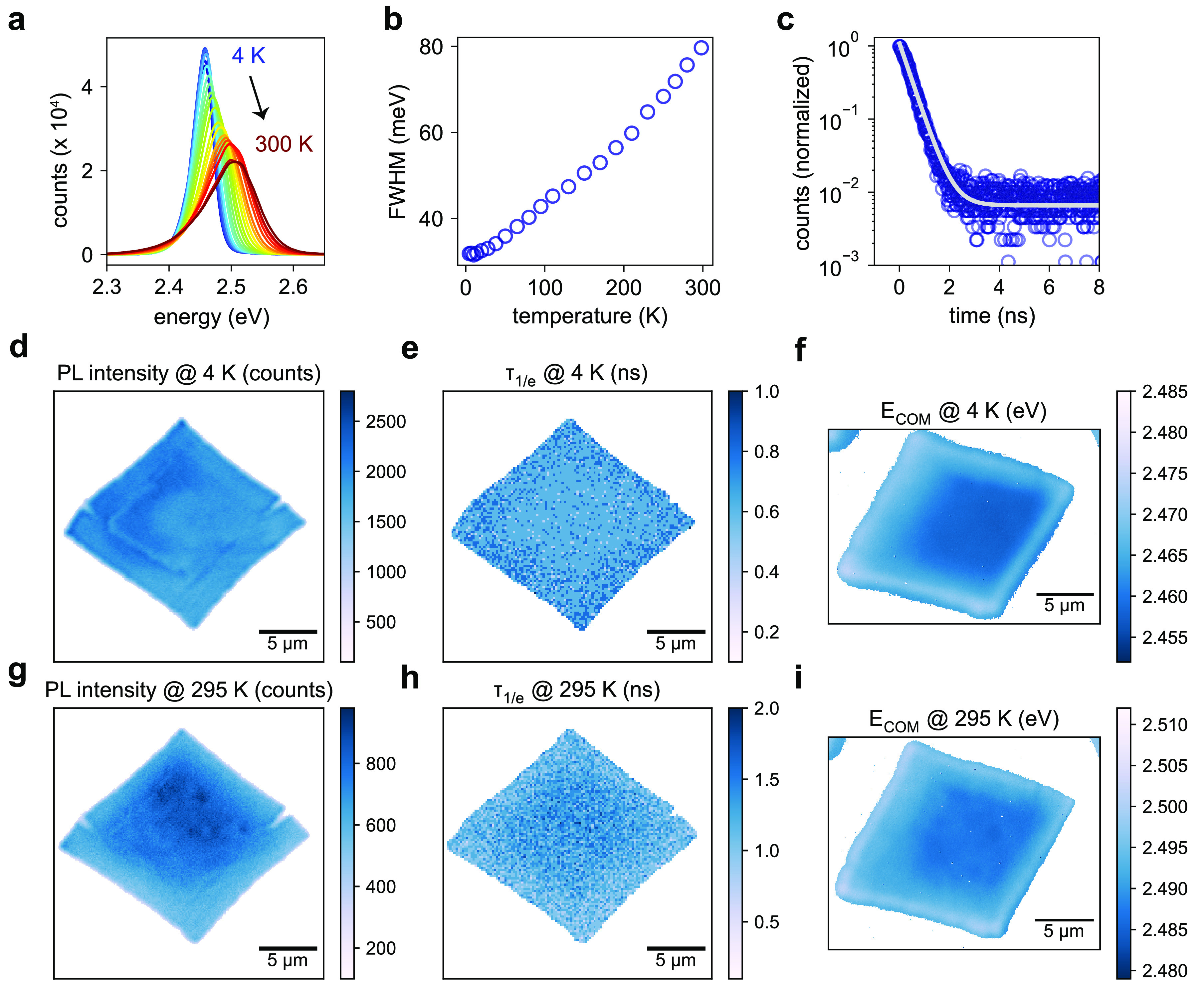
Narrow emission in strongly confined QD SLs. (a) Temperature-dependent PL spectra of a SL of 5 nm QDs with rhombic shape. (b) PL fwhm as a function of temperature. (c) Time-resolved PL of a QD SL at 4 K (blue circles) and double-exponential fit (gray line), revealing a short lifetime of ∼0.4 ns. (d–i) PL microscopy images of a single SL at 4 K (d–f) and 295 K (g–i), revealing a high spatial uniformity in the PL intensity (d,g), 1/e PL lifetime (τ_1/*e*_) (e,h), and center-of-mass energy *E*_COM_ (f,i), respectively.

Figure 4 explores the luminescent properties of QD SLs and possible benefits resulting from the high monodispersity and high degree of structural order. Figure 4a displays the temperature-dependent PL spectra of a single QD SL from 4 to 300 K. Defying the odds of the strong confinement regime, the SL displays narrow-band PL at 4 K (fwhm ∼33 meV, see Figure 4b), attesting a small inhomogeneous broadening contribution, in line with the high size and shape monodispersity (see Figure 1b). Upon increasing the temperature, the emission broadens, due to coupling of the excitons to thermally activated phonons. At room temperature, exciton–phonon coupling is the dominant contribution, with a QD SL line width (∼80 meV, see Figure 4b) quasi-identical with the single-QD line width (see Figure 3a). The low-temperature PL decay features an average time constant of ∼0.4 ns (see Figure 4c), identical, within experimental error, to the single-QD lifetime.

Spatially resolved confocal PL images (see Figures 4d and 4g) show intensity variations across the sample of well below 20% (standard deviation), consistent with the uniform SL thickness inferred from AFM (see Figure 2c) and SEM (see Figure 2b). Likewise, confocal PL images reveal a spatially uniform PL lifetime, with a minor increase toward the SL edge at 4 K (see Figures 4e) and a minor decrease at 295 K (see Figure 4h). However, both at 4 K (see Figure 4f) and 295 K (see Figure 4i), spatially resolved widefield hyperspectral PL images consistently reveal a slight increase of the center-of-mass PL energy *E*_COM_ by about 10–15 meV from the SL center toward the edge, qualitatively and quantitatively similar to large-QD SLs.^64^ In the latter report, the combined observation of a spectral blue-shift and a decreasing PL lifetime toward the SL edge was attributed to a loss in structural coherence, an increasing atomic misalignment between adjacent QDs, and growing compressive strain near the SL edge. While our room-temperature measurements would, in principle, be consistent with such a model, the lack of a decreased PL lifetime in edge-near regions at 4 K is inconsistent with strain as the origin for the blue-shift at SL edges. We suggest that elucidating the precise relationship between SL strain and PL properties (here: spectrum and lifetime) may require the careful exclusion of several alternative potential origins, *e.g.*, size and shape segregation within the SL,^64^ self-absorption,^65−67^ exciton diffusion,^68−71^ and photon propagation and recycling effects.^72−74^

## Conclusions

In conclusion, via a postsynthetic treatment employing DDAB ligands, we demonstrate chemically stable, strongly confined 5 nm CsPbBr_3_ QDs with cyan emission and well-defined absorption features. The high monodispersity in size and shape allows these QDs to self-assemble into SLs of exceptional long-range order. SLs exhibit an unusual rhombic macroscopic shape with an obtuse angle of about 104°, traced back to a C-centered orthorhombic QD packing. Likely, this unusual dense packing of cuboidal QDs is facilitated by the soft ligand shell which, unlike for larger QDs, represents a much higher volume fraction. Finally, we demonstrate that single 5 nm CsPbBr_3_ QDs make for good solution-processed room-temperature single-photon sources, with rapid radiative decay at cryogenic temperature, while SLs represent well-defined collective emitters with narrow emission bands and uniform spectral and intensity characteristics.

## Methods

### Safety Statement

No unexpected or unusually high safety hazards were encountered.

### Synthesis of CsPbBr_3_ QDs with a Size of 5 nm

We synthesized CsPbBr_3_ QDs adapting a synthesis procedure described by Dong et al.^9^

#### Cesium Oleate 0.16 M in Octadecene (ODE)

Cs_2_CO_3_ (250 mg, 0.77 mmol), oleic acid (0.8 mL), and ODE (8.8 mL) were mixed in a 25 mL flask. The mixture was degassed three times and then heated to 100–120 °C under N_2_ until it became clear. Cesium oleate in ODE was stored in the glovebox.

#### Synthesis of QDs

PbBr_2_ (75 mg, 0.2 mmol), ZnBr_2_ (Alfa Aesar, 180 mg, 0.8 mmol), and distilled mesitylene (5 mL) were mixed in a 25 mL flask under N_2_, stirring at 1400 rpm. The mixture was heated to 120 °C; distilled oleylamine (2 mL) and dried oleic acid (2 mL) were injected. The mixture was heated to 145 °C; 0.4 mL cesium oleate was injected from a 0.5 mL glass syringe. The reaction was quenched after 15 s with an ice bath.

#### Size Selection and Washing

The crude sample was centrifuged 3 min at 12,100 rpm; the precipitate was discarded. Twenty-seven mL ethyl acetate was added to the supernatant, and then it was centrifuged 5 min at 12,100 rpm, the supernatant was discarded, and the precipitate was dispersed in 1 mL anhydrous toluene

#### Postsynthesis Treatment

One mL QDs in toluene were mixed with 100 μL 0.01 M didodecyldimethylammonium bromide (DDAB) in toluene and stirred for 1 h. To obtain 0.01 M DDAB in toluene, 9.2 mg DDAB were dissolved in 2 mL anhydrous toluene.

### Self-Assembly of CsPbBr_3_ QDs

The SLs were prepared on the square 5 mm × 5 mm silicon substrates. Shortly before the self-assembly process, the silicon substrate was dipped into 4% solution of HF in water for 5 min, followed by intensive washing with deionized water. In a typical assembly process, the substrate was placed in a 10 mm × 10 mm × 10 mm Teflon well and 7 μL of purified QD solution in toluene or octane with concentration 1–1.2 mg/mL were spread onto the substrate. The well was covered with a glass slide to allow slow evaporation of the solvent. 3D SLs of CsPbBr_3_ QDs were formed upon complete evaporation of the toluene. Typical lateral dimensions of SLs ranged from 5 to 30 μm.

### Electron Microscopy Characterization

TEM and HAADF-STEM images as well as wide-angle electron diffraction (ED) patterns were collected using a JEOL JEM2200FS microscope operating at 200 kV accelerating voltage. High-resolution HAADF-STEM and HAADF-STEM images at different tilt angles were recorded using an FEI Titan Themis microscope operated at 300 kV with the aid of a motorized dual-axis tomography holder. TEM and ED images were compared with those simulated in Crystal Maker 10.4.5 and Single Crystal 3.1.5 software (purchased from CrystalMaker Software). SEM images were obtained on a FEI Helios 660 operated at 3 kV using immersion mode.

### Optical Microscopy and Spectroscopy at the Ensemble Level

Optical images were obtained using optical microscope Leica DM4M under UV-light. To assess the macroscopic shape of the SL as observed in optical microscopy (viewing direction perpendicular to the substrate) and extract quantitative information on the exposed SL angles (see histogram in Figure 2a), a custom image-recognition code was implemented, as described in more detail further below. Optical absorption spectra were measured with a Jasco V770 spectrometer in transmission mode. Photoluminescence spectra were measured in a 90° configuration using a Horiba Fluoromax-4P+ equipped with a photomultiplier tube and a monochromatized 150-W xenon lamp as an excitation source. The photoluminescence quantum yield of the samples was measured in a Hamamatsu Quantaurus-QY Plus UV–NIR absolute photoluminescence spectrometer (C13534–11) equipped with an integrating sphere.

### Synchrotron-Based SAXS and GISAXS

SAXS and GISAXS data were recorded at the AustroSAXS beamline of the Elettra Synchrotron (Trieste, IT).^75^ Measurements were performed at 8 keV X-ray energy, whereas images were collected using a large-area PILATUS3 1 M detector (DECTRIS Ltd., CH). The sample–detector distance was adjusted to 0.78 m for SAXS and to 1.8 m for GISAXS measurements and calibrated using Ag-behenate (*d*-spacing of 5.838 nm). Utilizing the typical convention for the momentum transfer, *i.e.*, *Q* = 4π sin(θ)/λ, where 2θ is the scattering angle and λ is the photon wavelength, this experimental geometry resulted in an accessible SAXS *Q*-range between 0.012–0.95 Å^–1^ and an accessible GISAXS *Q*-range of *Q*_V_ < 0.34 Å^–1^ and −0.12 < *Q*_H_ < 0.24 Å^–1^. SAXS measurements of QD dispersions were performed in a 1.5 mm quartz capillary. Sample and background measurements consisted of at least 15 exposures of 5 s each, to check for possible radiation damage. Each scattering image was azimuthally integrated using the SAXSDOG software package^76^ and subsequently corrected for primary-beam fluctuations and sample transmission, allowing adequate subsequent subtraction of the background signal.

The final scattering pattern was then fitted using an analytical model, assuming an orthogonal parallelepiped shape with Gaussian volume distribution. For a detailed model explanation, we refer to the section “SAXS model fitting” in the Supporting Information.

GISAXS images were taken at an incidence angle of 0.20° (aligned using position of the specular reflection), using an exposure time of ten seconds per image. For better statistics, the sample was horizontally scanned in steps of 0.5 mm, whereas images were subsequently averaged after correction for fluctuations in the primary beam intensity. The final images were converted to *Q*-space using the GIXSGUI software package^77^ and horizontal cuts to extract in-plane scattering patterns at the *L* = 0 and *L* = 1 order were extracted using NIKA2D.^78^ The positions of the SL reflections on the scattering images were calculated using GIXSGUI.^77^

### Synchrotron-Based WAXTS

X-ray total scattering measurements on CsPbBr_3_ QDs were performed at the X04SA-MS beamline of the Swiss Light Source (Paul Scherrer Institute, Villigen, CH),^78^ by filling a 0.5 mm borosilicate glass capillary of certified composition (Hilgenberg GmbH G50) with a toluene colloidal suspension of QDs.

A beam energy of 22 keV was set, and the operational wavelength (0.564513 Å) was accurately determined using a silicon powder standard (NIST 640d, a_0 = 0.543123(8) nm at 22.5 °C). Data were collected in the 0.4°–130° 2θ range using a single-photon counting silicon microstrip detector (MYTHEN II).^79^ Scattering from the sample, from the empty glass capillary, and from pure toluene were independently collected under the same experimental conditions.

Angle-dependent intensity corrections were applied to the raw data to account for sample attenuation due to absorption effects; sample absorption curves were determined using an X-ray tracing method^80^ and by measuring the transmitted beam from the filled capillary at room temperature, while for the empty capillary the X-ray attenuation coefficient was computed using its nominal composition. Angular calibrations were applied to the zero angle and *x*, *y* capillary offsets, derived from the certified silicon powder standard (NIST 640d) using locally developed procedures. Background and (absorption-corrected) capillary scattering contributions were subtracted from the sample signal, while the toluene scattering trace was added to the CsPbBr_3_ QD Debye scattering equation (DSE) model as a blank trace, suitably rescaled by linear least-squares.

WAXTS data were fitted using the DSE method utilizing atomistic models of the 5 nm CsPbBr_3_ QD, as described in detail in the Supporting Information section “The Debye Scattering Equation (DSE) Method”.

### Single-QD PL Spectroscopy

For single-QD PL spectroscopy, films of 5 nm CsPbBr_3_ QDs in a polystyrene matrix on glass substrate were prepared in a N_2_-filled glovebox. Briefly, a colloidal dispersion of CsPbBr_3_ QDs (1 mg/mL) was first diluted by a factor 100 in dry toluene (ACROS, 99.85%, extra dry, over molecular sieves), then by another factor of 100 in a dry toluene solution containing 1 mass % polystyrene (Aldrich, ∼280,000 MW). Subsequently, about 50 μL of the obtained dilute dispersion of QDs was spin-coated at 3000 rpm for 60 s onto a thin glass coverslip (Thorlabs, with a diameter of 25 mm, and a thickness of 170 ± 5 μm). The obtained QD film was placed onto a motorized precision *xyz*-stage with piezo drive (SmarAct GmbH) inside a home-built single-QD setup. The sample was excited by focusing the output of a fiber-coupled 405 nm pulsed laser (Picoquant, <50 ps pulses, 10 MHz repetition rate) onto the sample (1/e^2^ radius = 0.6 μm) using an oil-immersion objective (NA = 1.3, 100× magnification). According to the absorption cross section of 10^–14^ cm^2^ for 5 nm CsPbBr_3_ QDs reported by Maes et al.,^81^ the average number of excitons is estimated to be 0.2 per QD (at 10 μJ/cm^2^ excitation fluence). The emitted light is collected via the same objective and residual scattered excitation light is discarded via a dichroic beam splitter (Semrock, Di03-R442-t1-25x36) and a long-pass filter (Thorlabs, FELH0450). The emitted photons are then analyzed regarding their spectral distribution, using a spectrograph (Princeton Instruments, 0.5 m) and an electron-multiplied charge-coupled device (EMCCD, Princeton instruments) camera with a 1 s binning time, or, alternatively, regarding their photon statistics, using a Hanbury–Brown and Twiss (HBT) setup with a 50/50 beam splitter, two avalanche photodiodes (APDs, EXCELITAS, 250 ps temporal resolution) and a counting card for time-correlated single-photon counting (TCSPC, Picoquant).

For single QD measurements at cryogenic temperatures, the diluted dispersion of QDs were spin-coated onto a Si/SiO_2_ substrate (size around 0.5 cm × 0.5 cm), mounted on *xyz* nanopositioning stages inside an evacuated liquid-helium flow cryostat (Montana Instruments), and cooled to the targeted temperature of 4 K. The fiber-coupled 405 nm pulsed laser (20 MHz repetition rate) is focused (1/e^2^ radius = 1.2 μm) onto the sample by a microscope objective (NA = 0.8, 100× magnification). From the employed excitation fluence of 2.6 μJ/cm^2^, we estimate an average of about 0.05 generated excitons per QD. The emitted light is collected by the same objective and analyzed as in room temperature measurements.

### PL Spectroscopy of QD SLs

The time-resolved photoluminescence (TRPL) images at room temperature and 4 K were acquired under a vacuum using a constant-flow liquid helium cryostat (Oxford, MicroHires) with the sample located on the cryostat’s coldfinger. The cryostat was subsequently mounted on a confocal microscope setup (PicoQuant, MicroTime 200). An air objective (NA = 0.8, 100× magnification) was used to excite and collect emission from the sample (radius ∼1 μm). The excitation laser, a 405 nm pulsed diode (Picoquant, ∼100 ps, 0.5 MHz repetition rate, 8 nW), was directly focused onto the sample with an air objective. The emission signal was separated from the excitation light using a dichroic mirror (FF01-405/10, AHF/Semrock). A pinhole of 150 μm was included in the detection path, as well as an additional 425 nm long-pass filter (ET425LP, Chroma) to minimize the laser contribution to the recorded signal. The TRPL was then focused onto a Hybrid PMT detector connected to a PicoQuant acquisition card for time correlated single-photon counting (time resolution of 100 ps). Repetition rates of 0.5 MHz were used for the confocal maps.

Wide-field, hyperspectral microscopy measurements were carried out at room temperature and 4 K under a vacuum using a constant-flow liquid helium cryostat (Oxford, MicroHires) with the sample located on the cryostat’s coldfinger. The cryostat was subsequently mounted on a Photon etc. IMA system. A 405 nm continuous wave laser was used for luminescence excitation. The excitation laser was filtered by a dichroic mirror. The lamp light used for reflection measurements travels through the objective to the sample. The emitted light from the sample was incident on a volume Bragg grating, which splits the light spectrally onto a CCD camera. The detector was a 1040 × 1392 resolution silicon CCD camera kept at 0 °C with a thermoelectric cooler and has an operational wavelength range of 400–1000 nm. By scanning the angle of the grating relative to the incident light, the spectrum of light coming from each point on the sample was obtained.

## Supporting Information Available

Additional details on materials and methods, including superlattice shape analysis from optical microscopy images via an automated image recognition algorithm, analysis of SAXS and GISAXS data, fitting of WAXTS data via the Debye scattering equation method, as well as additional data, including AFM, SEM, and HAADF-STEM images, SAXS and GISAXS patterns, projected density of states via density functional theory, control of the QD packing via the ligand length, thermal broadening of single-QD PL spectra, and photostability of single QDs at cryogenic temperature (PDF)

## Notes

The DebUsSy program suite is freely available at https://debyeusersystem.github.io.

## References

[ref1] Protesescu L.; Yakunin S.; Bodnarchuk M. I.; Krieg F.; Caputo R.; Hendon C. H.; Yang R. X.; Walsh A.; Kovalenko M. V. Nanocrystals of cesium lead halide perovskites (CsPbX_3_, X = Cl, Br, and I): novel optoelectronic materials showing bright emission with wide color gamut. Nano Lett. 2015, 15 (6), 3692–3696. 10.1021/nl5048779. 25633588 PMC4462997

[ref2] Sercel P. C.; Lyons J. L.; Wickramaratne D.; Vaxenburg R.; Bernstein N.; Efros A. L. Exciton fine structure in perovskite nanocrystals. Nano Lett. 2019, 19 (6), 4068–4077. 10.1021/acs.nanolett.9b01467. 31088061

[ref3] Nguyen T. P. T.; Blundell S. A.; Guet C. One-photon absorption by inorganic perovskite nanocrystals: a theoretical study. Phys. Rev. B 2020, 101 (19), 195414 10.1103/PhysRevB.101.195414.

[ref4] Krieg F.; Sercel P. C.; Burian M.; Andrusiv H.; Bodnarchuk M. I.; Stöferle T.; Mahrt R. F.; Naumenko D.; Amenitsch H.; Rainò G.; et al. Monodisperse long-chain sulfobetaine-capped CsPbBr_3_ nanocrystals and their superfluorescent assemblies. ACS Cent. Sci. 2021, 7 (1), 135–144. 10.1021/acscentsci.0c01153. 33532576 PMC7845019

[ref5] Kovalenko M. V.; Protesescu L.; Bodnarchuk M. I. Properties and potential optoelectronic applications of lead halide perovskite nanocrystals. Science 2017, 358 (6364), 745–750. 10.1126/science.aam7093. 29123061

[ref6] Dey A.; Ye J.; De A.; Debroye E.; Ha S. K.; Bladt E.; Kshirsagar A. S.; Wang Z.; Yin J.; Wang Y.; et al. State of the art and prospects for halide perovskite nanocrystals. ACS Nano 2021, 15 (7), 10775–10981. 10.1021/acsnano.0c08903. 34137264 PMC8482768

[ref7] Shamsi J.; Rainò G.; Kovalenko M. V.; Stranks S. D. To nano or not to nano for bright halide perovskite emitters. Nat. Nanotechnol. 2021, 16 (11), 1164–1168. 10.1038/s41565-021-01005-z. 34759354

[ref8] John R. A.; Demirağ Y.; Shynkarenko Y.; Berezovska Y.; Ohannessian N.; Payvand M.; Zeng P.; Bodnarchuk M. I.; Krumeich F.; Kara G.; et al. Reconfigurable halide perovskite nanocrystal memristors for neuromorphic computing. Nat. Commun. 2022, 13 (1), 2074 10.1038/s41467-022-29727-1. 35440122 PMC9018677

[ref9] Dong Y. T.; Qiao T.; Kim D.; Parobek D.; Rossi D.; Son D. H. Precise control of quantum confinement in cesium lead halide perovskite quantum dots via thermodynamic equilibrium. Nano Lett. 2018, 18 (6), 3716–3722. 10.1021/acs.nanolett.8b00861. 29727576

[ref10] Qiao T.; Son D. H. Synthesis and properties of strongly quantum-confined cesium lead halide perovskite nanocrystals. Acc. Chem. Res. 2021, 54 (6), 1399–1408. 10.1021/acs.accounts.0c00706. 33566565

[ref11] Utzat H.; Sun W. W.; Kaplan A. E. K.; Krieg F.; Ginterseder M.; Spokoyny B.; Klein N. D.; Shulenberger K. E.; Perkinson C. F.; Kovalenko M. V.; et al. Coherent single-photon emission from colloidal lead halide perovskite quantum dots. Science 2019, 363 (6431), 1068 10.1126/science.aau7392. 30792359

[ref12] Becker M. A.; Scarpelli L.; Nedelcu G.; Rainò G.; Masia F.; Borri P.; Stoferle T.; Kovalenko M. V.; Langbein W.; Mahrt R. F. Long exciton dephasing time and coherent phonon coupling in cspbbr_2_cl perovskite nanocrystals. Nano Lett. 2018, 18 (12), 7546–7551. 10.1021/acs.nanolett.8b03027. 30407011

[ref13] Park Y.-S.; Guo S.; Makarov N. S.; Klimov V. I. Room temperature single-photon emission from individual perovskite quantum dots. ACS Nano 2015, 9 (10), 10386–10393. 10.1021/acsnano.5b04584. 26312994

[ref14] Rainò G.; Nedelcu G.; Protesescu L.; Bodnarchuk M. I.; Kovalenko M. V.; Mahrt R. F.; Stoferle T. Single cesium lead halide perovskite nanocrystals at low temperature: fast single-photon emission, reduced blinking, and exciton fine structure. ACS Nano 2016, 10 (2), 2485–2490. 10.1021/acsnano.5b07328. 26771336 PMC4768330

[ref15] Nagaoka Y.; Hills-Kimball K.; Tan R.; Li R.; Wang Z.; Chen O. Nanocube superlattices of cesium lead bromide perovskites and pressure-induced phase transformations at atomic and mesoscale levels. Adv. Mater. 2017, 29 (18), 1606666 10.1002/adma.201606666. 28295682

[ref16] van der Burgt J. S.; Geuchies J. J.; van der Meer B.; Vanrompay H.; Zanaga D.; Zhang Y.; Albrecht W.; Petukhov A. V.; Filion L.; Bals S.; et al. Cuboidal supraparticles self-assembled from cubic cspbbr_3_ perovskite nanocrystals. J. Phys. Chem. C 2018, 122 (27), 15706–15712. 10.1021/acs.jpcc.8b02699. PMC6143281 30245760

[ref17] Cherniukh I.; Rainò G.; Stöferle T.; Burian M.; Travesset A.; Naumenko D.; Amenitsch H.; Erni R.; Mahrt R. F.; Bodnarchuk M. I.; et al. Perovskite-type superlattices from lead halide perovskite nanocubes. Nature 2021, 593 (7860), 535–542. 10.1038/s41586-021-03492-5. 34040208

[ref18] Rainò G.; Becker M. A.; Bodnarchuk M. I.; Mahrt R. F.; Kovalenko M. V.; Stöferle T. Superfluorescence from lead halide perovskite quantum dot superlattices. Nature 2018, 563 (7733), 671–675. 10.1038/s41586-018-0683-0. 30405237

[ref19] Fakharuddin A.; Gangishetty M. K.; Abdi-Jalebi M.; Chin S.-H.; bin Mohd Yusoff A. R.; Congreve D. N.; Tress W.; Deschler F.; Vasilopoulou M.; Bolink H. J. Perovskite light-emitting diodes. Nat. Electron. 2022, 5 (4), 203–216. 10.1038/s41928-022-00745-7.

[ref20] Kim Y.-H.; Kim S.; Kakekhani A.; Park J.; Park J.; Lee Y.-H.; Xu H.; Nagane S.; Wexler R. B.; Kim D.-H.; et al. Comprehensive defect suppression in perovskite nanocrystals for high-efficiency light-emitting diodes. Nat. Photonics 2021, 15 (2), 148–155. 10.1038/s41566-020-00732-4.

[ref21] Hassan Y.; Park J. H.; Crawford M. L.; Sadhanala A.; Lee J.; Sadighian J. C.; Mosconi E.; Shivanna R.; Radicchi E.; Jeong M.; et al. Ligand-engineered bandgap stability in mixed-halide perovskite LEDs. Nature 2021, 591 (7848), 72–77. 10.1038/s41586-021-03217-8. 33658694

[ref22] Tong Y.; Yao E.-P.; Manzi A.; Bladt E.; Wang K.; Döblinger M.; Bals S.; Müller-Buschbaum P.; Urban A. S.; Polavarapu L.; et al. Spontaneous self-assembly of perovskite nanocrystals into electronically coupled supercrystals: toward filling the green gap. Adv. Mater. 2018, 30 (29), 1801117 10.1002/adma.201801117. 29870579

[ref23] Toso S.; Baranov D.; Giannini C.; Marras S.; Manna L. Wide-angle x-ray diffraction evidence of structural coherence in CsPbBr_3_ nanocrystal superlattices. ACS Mater. Lett. 2019, 1 (2), 272–276. 10.1021/acsmaterialslett.9b00217. 32954357 PMC7497715

[ref24] Cho K.; Yamada T.; Tahara H.; Tadano T.; Suzuura H.; Saruyama M.; Sato R.; Teranishi T.; Kanemitsu Y. Luminescence fine structures in single lead halide perovskite nanocrystals: size dependence of the exciton-phonon coupling. Nano Lett. 2021, 21 (17), 7206–7212. 10.1021/acs.nanolett.1c02122. 34415169

[ref25] Masada S.; Yamada T.; Tahara H.; Hirori H.; Saruyama M.; Kawawaki T.; Sato R.; Teranishi T.; Kanemitsu Y. Effect of A-site cation on photoluminescence spectra of single lead bromide perovskite nanocrystals. Nano Lett. 2020, 20 (5), 4022–4028. 10.1021/acs.nanolett.0c01417. 32330045

[ref26] Yazdani N.; Volk S.; Yarema O.; Yarema M.; Wood V. Size, ligand, and defect-dependent electron-phonon coupling in chalcogenide and perovskite nanocrystals and its impact on luminescence line widths. ACS Photonics 2020, 7 (5), 1088–1095. 10.1021/acsphotonics.0c00034.

[ref27] Ramade J.; Andriambariarijaona L. M.; Steinmetz V.; Goubet N.; Legrand L.; Barisien T.; Bernardot F.; Testelin C.; Lhuillier E.; Bramati A.; Chamarro M. Exciton-phonon coupling in a CsPbBr_3_ single nanocrystal. Appl. Phys. Lett. 2018, 112 (7), 072104 10.1063/1.5018413. 29560979

[ref28] Iaru C. M.; Geuchies J. J.; Koenraad P. M.; Vanmaekelbergh D.; Silov A. Y. Strong carrier-phonon coupling in lead halide perovskite nanocrystals. ACS Nano 2017, 11 (11), 11024–11030. 10.1021/acsnano.7b05033. 29019652 PMC5707632

[ref29] Mondal A.; Aneesh J.; Kumar Ravi V.; Sharma R.; Mir W. J.; Beard M. C.; Nag A.; Adarsh K. V. Ultrafast exciton many-body interactions and hot-phonon bottleneck in colloidal cesium lead halide perovskite nanocrystals. Phys. Rev. B 2018, 98 (11), 115418 10.1103/PhysRevB.98.115418.

[ref30] Yazdani N.; Bodnarchuk M. I.; Bertolotti F.; Masciocchi N.; Fureraj I.; Guzelturk B.; Cotts B. L.; Zajac M.; Rainò G.; Jansen M.; Phonon-mediated attractive interactions between excitons in lead-halide-perovskites. arXiv, March 11, 2022, 2203.06286. 10.48550/arXiv.2203.06286 (accessed November 12, 2022). PMC10791581 38261834

[ref31] Zasedatelev A. V.; Baranikov A. V.; Sannikov D.; Urbonas D.; Scafirimuto F.; Shishkov V. Y.; Andrianov E. S.; Lozovik Y. E.; Scherf U.; Stöferle T.; et al. Single-photon nonlinearity at room temperature. Nature 2021, 597 (7877), 493–497. 10.1038/s41586-021-03866-9. 34552252

[ref32] Swift M. W.; Lyons J. L.; Efros A. L.; Sercel P. C. Rashba exciton in a 2D perovskite quantum dot. Nanoscale 2021, 13 (39), 16769–16780. 10.1039/D1NR04884H. 34604886

[ref33] Becker M. A.; Vaxenburg R.; Nedelcu G.; Sercel P. C.; Shabaev A.; Mehl M. J.; Michopoulos J. G.; Lambrakos S. G.; Bernstein N.; Lyons J. L.; et al. Bright triplet excitons in caesium lead halide perovskites. Nature 2018, 553 (7687), 189 10.1038/nature25147. 29323292

[ref34] Tamarat P.; Bodnarchuk M. I.; Trebbia J. B.; Erni R.; Kovalenko M. V.; Even J.; Lounis B. The ground exciton state of formamidinium lead bromide perovskite nanocrystals is a singlet dark state. Nat. Mater. 2019, 18 (7), 717–724. 10.1038/s41563-019-0364-x. 31086320

[ref35] Bodnarchuk M. I.; Boehme S. C.; ten Brinck S.; Bernasconi C.; Shynkarenko Y.; Krieg F.; Widmer R.; Aeschlimann B.; Gunther D.; Kovalenko M. V.; et al. Rationalizing and controlling the surface structure and electronic passivation of cesium lead halide nanocrystals. ACS Energy Lett. 2019, 4 (1), 63–74. 10.1021/acsenergylett.8b01669. 30662955 PMC6333230

[ref36] Stelmakh A.; Aebli M.; Baumketner A.; Kovalenko M. V. On the mechanism of alkylammonium ligands binding to the surface of CsPbBr_3_ nanocrystals. Chem. Mater. 2021, 33 (15), 5962–5973. 10.1021/acs.chemmater.1c01081. 34393361 PMC8359008

[ref37] Bertolotti F.; Protesescu L.; Kovalenko M. V.; Yakunin S.; Cervellino A.; Billinge S. J. L.; Terban M. W.; Pedersen J. S.; Masciocchi N.; Guagliardi A. Coherent nanotwins and dynamic disorder in cesium lead halide perovskite nanocrystals. ACS Nano 2017, 11 (4), 3819–3831. 10.1021/acsnano.7b00017. 28394579 PMC5800404

[ref38] Bertolotti F.; Nedelcu G.; Vivani A.; Cervellino A.; Masciocchi N.; Guagliardi A.; Kovalenko M. V. Crystal structure, morphology, and surface termination of cyan-emissive, six-monolayers-thick cspbbr_3_ nanoplatelets from x-ray total scattering. ACS Nano 2019, 13 (12), 14294–14307. 10.1021/acsnano.9b07626. 31747248 PMC6933817

[ref39] Brennan M. C.; Toso S.; Pavlovetc I. M.; Zhukovskyi M.; Marras S.; Kuno M.; Manna L.; Baranov D. Superlattices are greener on the other side: how light transforms self-assembled mixed halide perovskite nanocrystals. ACS Energy Lett. 2020, 5 (5), 1465–1473. 10.1021/acsenergylett.0c00630.

[ref40] Travesset A. Soft skyrmions, spontaneous valence and selection rules in nanoparticle superlattices. ACS Nano 2017, 11 (6), 5375–5382. 10.1021/acsnano.7b02219. 28514592

[ref41] Travesset A. Topological structure prediction in binary nanoparticle superlattices. Soft Matter 2017, 13 (1), 147–157. 10.1039/C6SM00713A. 27156535

[ref42] Huang X.; Zhu J.; Ge B.; Deng K.; Wu X.; Xiao T.; Jiang T.; Quan Z.; Cao Y. C.; Wang Z. Understanding Fe_3_O_4_ nanocube assembly with reconstruction of a consistent superlattice phase diagram. J. Am. Chem. Soc. 2019, 141 (7), 3198–3206. 10.1021/jacs.8b13082. 30685973

[ref43] Zhang J.; Zhu J.; Li R.; Fang J.; Wang Z. Entropy-driven Pt_3_Co nanocube assembles and thermally mediated electrical conductivity with anisotropic variation of the rhombohedral superlattice. Nano Lett. 2017, 17 (1), 362–367. 10.1021/acs.nanolett.6b04295. 27936796

[ref44] Quan Z.; Loc W. S.; Lin C.; Luo Z.; Yang K.; Wang Y.; Wang H.; Wang Z.; Fang J. Tilted face-centered-cubic supercrystals of PbS nanocubes. Nano Lett. 2012, 12 (8), 4409–4413. 10.1021/nl302324b. 22813064

[ref45] Li R.; Bian K.; Wang Y.; Xu H.; Hollingsworth J. A.; Hanrath T.; Fang J.; Wang Z. An obtuse rhombohedral superlattice assembled by Pt nanocubes. Nano Lett. 2015, 15 (9), 6254–6260. 10.1021/acs.nanolett.5b02879. 26280872

[ref46] Yarita N.; Tahara H.; Ihara T.; Kawawaki T.; Sato R.; Saruyama M.; Teranishi T.; Kanemitsu Y. Dynamics of charged excitons and biexcitons in CsPbBr_3_ perovskite nanocrystals revealed by femtosecond transient-absorption and single-dot luminescence spectroscopy. J. Phys. Chem. Lett. 2017, 8 (7), 1413–1418. 10.1021/acs.jpclett.7b00326. 28286951

[ref47] Hu F.; Zhang H.; Sun C.; Yin C.; Lv B.; Zhang C.; Yu W. W.; Wang X.; Zhang Y.; Xiao M. Superior optical properties of perovskite nanocrystals as single photon emitters. ACS Nano 2015, 9 (12), 12410–12416. 10.1021/acsnano.5b05769. 26522082

[ref48] Pierini S.; D’Amato M.; Goyal M.; Glorieux Q.; Giacobino E.; Lhuillier E.; Couteau C.; Bramati A. Highly photostable perovskite nanocubes: toward integrated single photon sources based on tapered nanofibers. ACS Photonics 2020, 7 (8), 2265–2272. 10.1021/acsphotonics.0c00820.

[ref49] Zhu C.; Marczak M.; Feld L.; Boehme S. C.; Bernasconi C.; Moskalenko A.; Cherniukh I.; Dirin D.; Bodnarchuk M. I.; Kovalenko M. V.; et al. Room-temperature, highly pure single-photon sources from all-inorganic lead halide perovskite quantum dots. Nano Lett. 2022, 22 (9), 3751–3760. 10.1021/acs.nanolett.2c00756. 35467890 PMC9101069

[ref50] Schmitz A.; Montanarella F.; Schaberg L. L.; Abdelbaky M.; Kovalenko M. V.; Bacher G. Optical probing of crystal lattice configurations in single cspbbr_3_ nanoplatelets. Nano Lett. 2021, 21 (21), 9085–9092. 10.1021/acs.nanolett.1c02775. 34672607

[ref51] Pashaei Adl H.; Gorji S.; Muñoz-Matutano G.; Sánchez-Alarcón R. I.; Abargues R.; Gualdrón-Reyes A. F.; Mora-Seró I.; Martínez-Pastor J. P. Homogeneous and inhomogeneous broadening in single perovskite nanocrystals investigated by micro-photoluminescence. J. Lumin. 2021, 240, 118453 10.1016/j.jlumin.2021.118453.

[ref52] Raino G.; Yazdani N.; Boehme S. C.; Kober-Czerny M.; Zhu C.; Krieg F.; Rossell M. D.; Erni R.; Wood V.; Infante I.; Kovalenko M. V. Ultra-narrow room-temperature emission from single CsPbBr_3_ perovskite quantum dots. Nat. Commun. 2022, 13 (1), 2587 10.1038/s41467-022-30016-0. 35546149 PMC9095639

[ref53] Palstra I. M.; de Buy Wenniger I. M.; Patra B. K.; Garnett E. C.; Koenderink A. F. Intermittency of CsPbBr_3_ perovskite quantum dots analyzed by an unbiased statistical analysis. J. Phys. Chem. C 2021, 125 (22), 12061–12072. 10.1021/acs.jpcc.1c01671. PMC8282187 34276863

[ref54] Seth S.; Ahmed T.; Samanta A. Photoluminescence flickering and blinking of single CsPbBr_3_ perovskite nanocrystals: revealing explicit carrier recombination dynamics. J. Phys. Chem. Lett. 2018, 9 (24), 7007–7014. 10.1021/acs.jpclett.8b02979. 30500204

[ref55] Palstra I. M.; Koenderink A. F. A Python toolbox for unbiased statistical analysis of fluorescence intermittency of multilevel emitters. J. Phys. Chem. C 2021, 125 (22), 12050–12060. 10.1021/acs.jpcc.1c01670. PMC8282189 34276862

[ref56] Isarov M.; Tan L. Z.; Bodnarchuk M. I.; Kovalenko M. V.; Rappe A. M.; Lifshitz E. Rashba effect in a single colloidal CsPbBr_3_ perovskite nanocrystal detected by magneto-optical measurements. Nano Lett. 2017, 17 (8), 5020–5026. 10.1021/acs.nanolett.7b02248. 28657325

[ref57] Labeau O.; Tamarat P.; Lounis B. Temperature dependence of the luminescence lifetime of single CdSe/ZnS quantum dots. Phys. Rev. Lett. 2003, 90 (25), 257404 10.1103/PhysRevLett.90.257404. 12857165

[ref58] Biadala L.; Louyer Y.; Tamarat P.; Lounis B. Direct observation of the two lowest exciton zero-phonon lines in single CdSe/ZnS nanocrystals. Phys. Rev. Lett. 2009, 103 (3), 037404 10.1103/PhysRevLett.103.037404. 19659317

[ref59] Schaller R. D.; Crooker S. A.; Bussian D. A.; Pietryga J. M.; Joo J.; Klimov V. I. Revealing the exciton fine structure of PbSe nanocrystal quantum dots using optical spectroscopy in high magnetic fields. Phys. Rev. Lett. 2010, 105 (6), 067403 10.1103/PhysRevLett.105.067403. 20868011

[ref60] Biadala L.; Siebers B.; Beyazit Y.; Tessier M. D.; Dupont D.; Hens Z.; Yakovlev D. R.; Bayer M. Band-edge exciton fine structure and recombination dynamics in InP/ZnS colloidal nanocrystals. ACS Nano 2016, 10 (3), 3356–3364. 10.1021/acsnano.5b07065. 26889780

[ref61] Fu M.; Tamarat P.; Trebbia J. B.; Bodnarchuk M. I.; Kovalenko M. V.; Even J.; Lounis B. Unraveling exciton-phonon coupling in individual FAPbI_3_ nanocrystals emitting near-infrared single photons. Nat. Commun. 2018, 9 (1), 3318 10.1038/s41467-018-05876-0. 30127339 PMC6102301

[ref62] Iaru C. M.; Brodu A.; van Hoof N. J. J.; ter Huurne S. E. T.; Buhot J.; Montanarella F.; Buhbut S.; Christianen P. C. M.; Vanmaekelbergh D.; de Mello Donega C.; et al. Fröhlich interaction dominated by a single phonon mode in CsPbBr_3_. Nat. Commun. 2021, 12 (1), 5844 10.1038/s41467-021-26192-0. 34615880 PMC8494801

[ref63] Boehme S. C.; Brinck S. t.; Maes J.; Yazdani N.; Zapata F.; Chen K.; Wood V.; Hodgkiss J. M.; Hens Z.; Geiregat P.; et al. Phonon-mediated and weakly size-dependent electron and hole cooling in CsPbBr_3_ nanocrystals revealed by atomistic simulations and ultrafast spectroscopy. Nano Lett. 2020, 20 (3), 1819–1829. 10.1021/acs.nanolett.9b05051. 32049539 PMC7997624

[ref64] Lapkin D.; Kirsch C.; Hiller J.; Andrienko D.; Assalauova D.; Braun K.; Carnis J.; Kim Y. Y.; Mandal M.; Maier A.; et al. Spatially resolved fluorescence of caesium lead halide perovskite supercrystals reveals quasi-atomic behavior of nanocrystals. Nat. Commun. 2022, 13 (1), 892 10.1038/s41467-022-28486-3. 35173165 PMC8850480

[ref65] Di Stasio F.; Imran M.; Akkerman Q. A.; Prato M.; Manna L.; Krahne R. Reversible concentration-dependent photoluminescence quenching and change of emission color in CsPbBr_3_ nanowires and nanoplatelets. J. Phys. Chem. Lett. 2017, 8 (12), 2725–2729. 10.1021/acs.jpclett.7b01305. 28581755

[ref66] Meinardi F.; Akkerman Q. A.; Bruni F.; Park S.; Mauri M.; Dang Z.; Manna L.; Brovelli S. Doped halide perovskite nanocrystals for reabsorption-free luminescent solar concentrators. ACS Energy Lett. 2017, 2 (10), 2368–2377. 10.1021/acsenergylett.7b00701. 31206029 PMC6559125

[ref67] van der Laan M.; de Weerd C.; Poirier L.; van de Water O.; Poonia D.; Gomez L.; Kinge S.; Siebbeles L. D. A.; Koenderink A. F.; Gregorkiewicz T.; et al. Photon recycling in CsPbBr_3_ all-inorganic perovskite nanocrystals. ACS Photonics 2021, 8 (11), 3201–3208. 10.1021/acsphotonics.1c00953. 34820474 PMC8603385

[ref68] Penzo E.; Loiudice A.; Barnard E. S.; Borys N. J.; Jurow M. J.; Lorenzon M.; Rajzbaum I.; Wong E. K.; Liu Y.; Schwartzberg A. M.; et al. Long-range exciton diffusion in two-dimensional assemblies of cesium lead bromide perovskite nanocrystals. ACS Nano 2020, 14 (6), 6999–7007. 10.1021/acsnano.0c01536. 32459460

[ref69] Baldwin A.; Delport G.; Leng K.; Chahbazian R.; Galkowski K.; Loh K. P.; Stranks S. D. Local energy landscape drives long-range exciton diffusion in two-dimensional halide perovskite semiconductors. J. Phys. Chem. Lett. 2021, 12 (16), 4003–4011. 10.1021/acs.jpclett.1c00823. 33877840 PMC8154849

[ref70] Giovanni D.; Righetto M.; Zhang Q.; Lim J. W. M.; Ramesh S.; Sum T. C. Origins of the long-range exciton diffusion in perovskite nanocrystal films: photon recycling vs exciton hopping. Light: Sci. Appl. 2021, 10 (1), 2 10.1038/s41377-020-00443-z. 33386385 PMC7775951

[ref71] Luo Y.; Zhou S.; Dang Z.; Gao P. Probing the exciton diffusion length of short-ligands passivated metal halide perovskite nanocrystal films. J. Phys. Chem. C 2021, 125 (50), 27638–27646. 10.1021/acs.jpcc.1c07830.

[ref72] Dursun I.; Zheng Y.; Guo T.; De Bastiani M.; Turedi B.; Sinatra L.; Haque M. A.; Sun B.; Zhumekenov A. A.; Saidaminov M. I.; et al. Efficient photon recycling and radiation trapping in cesium lead halide perovskite waveguides. ACS Energy Lett. 2018, 3 (7), 1492–1498. 10.1021/acsenergylett.8b00758.

[ref73] Gan Z.; Chen W.; Yuan L.; Cao G.; Zhou C.; Huang S.; Wen X.; Jia B. External Stokes shift of perovskite nanocrystals enlarged by photon recycling. Appl. Phys. Lett. 2019, 114 (1), 011906 10.1063/1.5081805.

[ref74] Yamada T.; Yamada Y.; Kanemitsu Y. Photon recycling in perovskite CH_3_NH_3_PbX_3_ (X = I, Br, Cl) bulk single crystals and polycrystalline films. J. Lumin. 2020, 220, 116987 10.1016/j.jlumin.2019.116987.

[ref75] Amenitsch H.; Bernstorff S.; Laggner P. High-flux beamline for small-angle x-ray scattering at ELETTRA. Rev. Sci. Instrum. 1995, 66 (2), 1624–1626. 10.1063/1.1145864.

[ref76] Burian M.; Meisenbichler C.; Naumenko D.; Amenitsch H. SAXSDOG: open software for real-time azimuthal integration of 2D scattering images. J. Appl. Crystallogr. 2022, 55 (3), 677–685. 10.1107/S1600576722003685. 35719301 PMC9172040

[ref77] Jiang Z. GIXSGUI: a MATLAB toolbox for grazing-incidence X-ray scattering data visualization and reduction, and indexing of buried three-dimensional periodic nanostructured films. J. Appl. Crystallogr. 2015, 48 (3), 917–926. 10.1107/S1600576715004434.

[ref78] Willmott P. R.; Meister D.; Leake S. J.; Lange M.; Bergamaschi A.; Boge M.; Calvi M.; Cancellieri C.; Casati N.; Cervellino A.; et al. The Materials Science beamline upgrade at the Swiss Light Source. J. Synchrotron Radiat. 2013, 20 (5), 667–682. 10.1107/S0909049513018475. 23955029 PMC3747948

[ref79] Bergamaschi A.; Cervellino A.; Dinapoli R.; Gozzo F.; Henrich B.; Johnson I.; Kraft P.; Mozzanica A.; Schmitt B.; Shi X. The MYTHEN detector for X-ray powder diffraction experiments at the Swiss Light Source. J. Synchrotron Radiat. 2010, 17 (5), 653–668. 10.1107/S0909049510026051. 20724787 PMC2924792

[ref80] Bowden M.; Ryan M. Absorption correction for cylindrical and annular specimens and their containers or supports. J. Appl. Crystallogr. 2010, 43 (4), 693–698. 10.1107/S0021889810021114.

[ref81] Maes J.; Balcaen L.; Drijvers E.; Zhao Q.; De Roo J.; Vantomme A.; Vanhaecke F.; Geiregat P.; Hens Z. Light absorption coefficient of CsPbBr_3_ perovskite nanocrystals. J. Phys. Chem. Lett. 2018, 9 (11), 3093–3097. 10.1021/acs.jpclett.8b01065. 29790351

