# Supplementary material for: Strongly Confined CsPbBr3 Quantum Dots as Quantum Emitters and Building Blocks for Rhombic Superlattices
Source: ACS Nano. 2023 Jan 31;17(3):2089–100. doi: 10.1021/acsnano.2c07677 (PMC9933619; doi:10.1021/acsnano.2c07677)
Supplement: Supplementary file 1 — nn2c07677_si_001.pdf [file nn2c07677_si_001.pdf]

Supporting Information

Strongly confined $CsPbBr_3$ quantum dots as quantum emitters and building blocks for rhombic superlattices

*Simon C. Boehme,[1,2#] Maryna I. Bodnarchuk,[1,2#] Max Burian,[3¶] Federica Bertolotti,[4] Ihor Cherniukh,[1,2] Caterina Bernasconi,[1,2] Chenglian Zhu,[1,2] Rolf Erni,[5] Heinz Amenitsch,[6] Denys Naumenko,[6] Hordii Andrusiv, [1,2] Nazar Semkiv, [1,2] Rohit Abraham John, [1,2] Alan Baldwin,[7,8] Krzysztof Galkowski,[7] Norberto Masciocchi,[4] Samuel D. Stranks,[7,8] Gabriele Rainò,[1,2] Antonietta Guagliardi,[9] Maksym V. Kovalenko[1,2]**

[1] Institute of Inorganic Chemistry, Department of Chemistry and Applied Biosciences, ETH Zürich, 8093 Zürich, Switzerland

[2] Laboratory for Thin Films and Photovoltaics, Empa, Swiss Federal Laboratories for Materials Science and Technology, 8600 Dübendorf, Switzerland

[3] Swiss Light Source, Paul Scherrer Institute, 5232 Villigen, Switzerland

[4] Department of Science and High Technology and To.Sca.Lab., University of Insubria, via Valleggio 11, 22100 Como, Italy

[5] Electron Microscopy Center, Empa, Swiss Federal Laboratories for Materials Science and Technology, 8600 Dübendorf, Switzerland

[6] Institute of Inorganic Chemistry, Graz University of Technology, 8010 Graz, Austria

[7] Cavendish Laboratory, University of Cambridge, JJ Thomson Avenue, Cambridge CB3 0HE, U.K.

[8] Department of Chemical Engineering & Biotechnology, University of Cambridge, Philippa Fawcett Drive, Cambridge CB3 0AS, U.K.

[9] Istituto di Cristallografia and To.Sca.Lab, Consiglio Nazionale delle Ricerche, via Valleggio 11, 22100 Como, Italy

[#]S.C.B. and M.I.B. contributed equally

SAXS model fitting

The scattering pattern was fitted using the analytical model used in F. Krieg et al.[11] In short, we describe the model scattering intensity as:

$$I_{mod}(q) = BG + I_0 \cdot \sum_{i}^{N_{SD}} G(r_i, a, \sigma) \cdot V \cdot F\left(q, r_i, \frac{b}{a}, \frac{c}{a}\right)$$

The fitting parameters are: (a) the first (smallest) NC side length, $(\frac{b}{a})$ the aspect ratio of the second to first NC side length, $(\frac{c}{a})$ the aspect ratio of the third to first NC side length, (σ) the standard-deviation of the size-distribution (relative) and (I_0) the forward scattering intensity scalar.[1] The effect of polydispersity is taken into account by performing the numerical average (summation) over the Gaussian distribution $G(r_i, a, \sigma)$, here consisting of N_{SD} = 51 discrete points, which is discretized over the range $a(1 - 3\sigma) < r_i < a(1 + 3\sigma)$ and is weighted by volume $V = r_i^3 \cdot \frac{b}{a} \cdot \frac{c}{a}$. The formfactor contribution stemming from the NC shape $F\left(q, r_i, \frac{b}{a}, \frac{c}{a}\right)$ is calculated using a theoretical orthorhombic model according to literature[2] such that $F(q = 0) = 1$, where r_i denotes the shortest NC side length corresponding to the respective size-distribution weight such that the other sides are scaled by $\frac{b}{a}$ and $\frac{c}{a}$.

The scattering pattern was fitted over the angular regime of 0.11 < q < 6.2 nm^{-1}, as shown in Figure 1b of the main text. During the fitting process, all parameters were optimized (see Table S1 for results) - only the background (BG) was kept fixed to mitigate potential cross-corelation uncertainties with other fitting variables. Overall, we find outstanding agreement of experimental and model scattering patterns, as quantitatively evidenced by the goodnesss-of-fit value $\chi^2 = 1.94$.

Table S1 | Fit results of the analytical SAXS model

	BG	I_0	a	b/a	c/a	σ	χ^2
	[.]	[.]	[nm]	[1]	[1]	[%]	[.]
**NC sample**	0.073	3‘081	4.7	1.19	1.23	7.5	**1.94**
**Error [%]**	-	1.2	2.8	3.7	3.9	24.0	

The Debye scattering equation (DSE) method

The DSE, employed for fitting the WAXTS data reported in the main text, provides the average differential elastic cross-section (or the powder diffraction pattern) of a randomly oriented powder from the distribution of interatomic distances between atomic pairs, without any assumption of periodicity and order:[3,4]

$$I(Q) = \sum_{j=1}^{N} f_j(Q)^2 o_j^2 + 2\sum_{j>i}^{N} f_j(Q) f_i(Q) T_j(Q) T_i(Q) o_j o_i \frac{\sin(Qd_{ij})}{(Qd_{ij})}$$

where $Q = 4\pi sin\theta/\lambda$ is the magnitude of the scattering vector, λ is the radiation wavelength, f_i is the atomic form factor of element i, d_{ij} is the interatomic distance between atoms i and j, N is the total number of atoms and T and o are the thermal atomic displacement parameter and the site occupancy factor associated to each atomic species, respectively. The first summation in the above equation includes the contributions of zero distances between one atom and itself and the second term (the interference term) the non-zero interatomic distances $d_{ij} = |r_i - r_j|$.

To compute the DSE according to the Debussy[5] strategy, a bivariate population of atomistic models of $CsPbBr_3$ QDs was generated, by packing the orthorhombic building block (in the *Pnma* setting) along three growth directions: one (independent) parallel to the b axis of the unit cell and the other two (characterized by an equal number of steps) obtained as a combination of **a** and **c** axes (**a**+**c** and **a-c**).

This model construction was selected to obtain a pseudocubic morphology for $CsPbBr_3$ QDs, exposing four {101} and two {010} facets (indices in the orthorhombic *Pnma* setting), in agreement with HRTEM observations, as detailed in the main text and in ref. 6. We note here that two {010} facets, *i.e.* 010 and 0-10, make the so called pinacoid, while the remaining {101} faces are the four equivalent lateral faces of a rhombic prism aligned in the b unit cell direction.

All six facets were terminated with CsBr moieties, the site occupancy of which are subsequently refined to adjust the QD surface termination against the experimental data.

The step between two consecutive clusters in the population, along each growth direction, was set to 5.88 Å (*i.e.* equivalent to the edge of a primitive cubic unit cell).

To improve the quality of the fit and to account for the apparent "cubicity" of the experimental WAXTS pattern (*i.e.* with practically absent orthorhombic superstructure peaks), slip planes were added to the QD models (one per each cluster of the population) according to the rule ½<101>(010) (in *Pnma* setting), as detailed in ref. 7.

The interatomic distances of these clusters are then computed, sampled (according to a Gaussian sampling strategy, to reduce the computational times),[8] and used to compute the model DSE.

A simultaneous DSE fit of the small-angle Porod region (0.21 $Å^{-1} \leq Q \leq$ 0.68 $Å^{-1}$), available from the synchrotron data collection, and of the WAXTS range (0.68 $Å^{-1} \leq Q \leq$ 14.31 $Å^{-1}$) has been performed using exactly the same atomistic and morphological model.

After the model optimization, an average stoichiometry Cs:Pb:Br = 1.0:1.0:3.2 was found, suggesting a slight excess of Br- anions on QDs facets (WAXTS goodness of fit = 1.64), with respect to the stoichiometric model (Cs:Pb:Br = 1.0:1.0:3.0; WAXTS goodness of fit = 1.66).

The average sample sizes and their distributions along the two independent growth directions were modeled using a bivariate log-normal distribution function, resulting in: (i) mass-weighted values, as provided in the main text, $L_a = L_c = 5.20$ nm ($\sigma/L = 0.08$), $L_b = 4.54$ nm ($\sigma/L = 0.17$), or (ii) number-based values, $L_a = L_c = 5.14$ nm ($\sigma/L = 0.08$), $L_b = 4.41$ ($\sigma/L = 0.17$).

Photoluminescence quantum yield

The photoluminescence quantum yield of our 5 nm CsPbBr3 QDs is about 65% in colloidal dispersion and above 50% in film.

Increase of the $CsPbBr_3$ band gap due to strong quantum confinement

At room temperature, the band gap of bulk $CsPbBr_3$ in absorption is about 2.38 eV (see *e.g. J. Phys. Chem. Lett. 2020, 11, 7, 2490–2496* and *Nano Lett. 2022, 22, 4, 1778–1785*). Considering the absorption band gap of 2.54 eV (488 nm) in our 5 nm $CsPbBr_3$ QDs, this translates into a confinement-induced blueshift of about 0.16 eV (= 2.54 eV – 2.38 eV).

Additional AFM images

Typical superlattices (SL) of 5 nm $CsPbBr_3$ quantum dots (QDs) exhibit thicknesses of several hundreds of nanometers, see Figure S1 for several representative SLs. The SL indicated by the cyan rectangle in Figure S1b corresponds to the SL shown in Figure 2c of the main text. This SL exhibits a thickness of about 0.7 µm and a root-mean-square roughness of 11.86 nm.

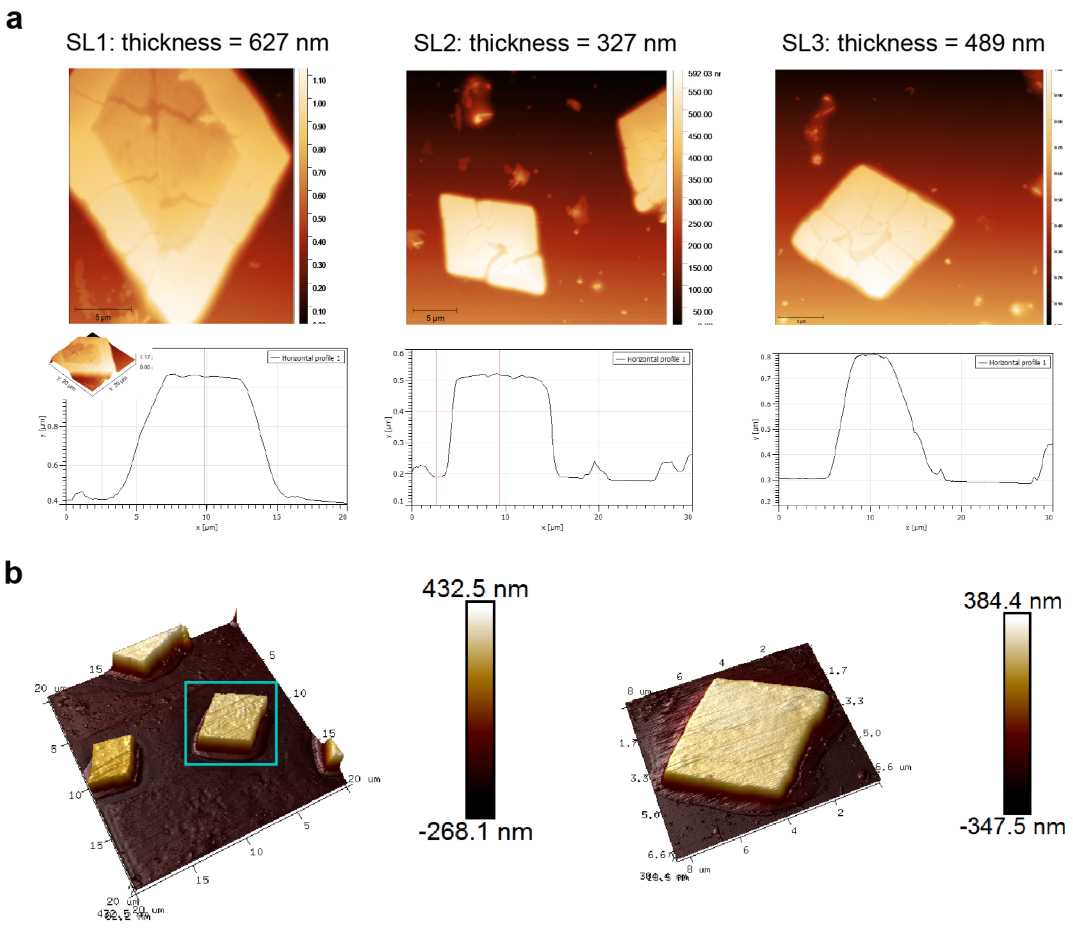

Figure S1 | AFM-derived SL thickness. (a) thickness maps (*upper panels*) and thickness profiles (*lower panels*) for three representative SLs; the average thickness is about 0.5 µm. (b) Larger-area thickness map; *left:* the SL shown in Figure 2c of the main text (indicated by the cyan rectangle) and its surrounding; *right:* a magnified view of the region indicated by the cyan rectangle (identical to Figure 2c).

Additional SEM images

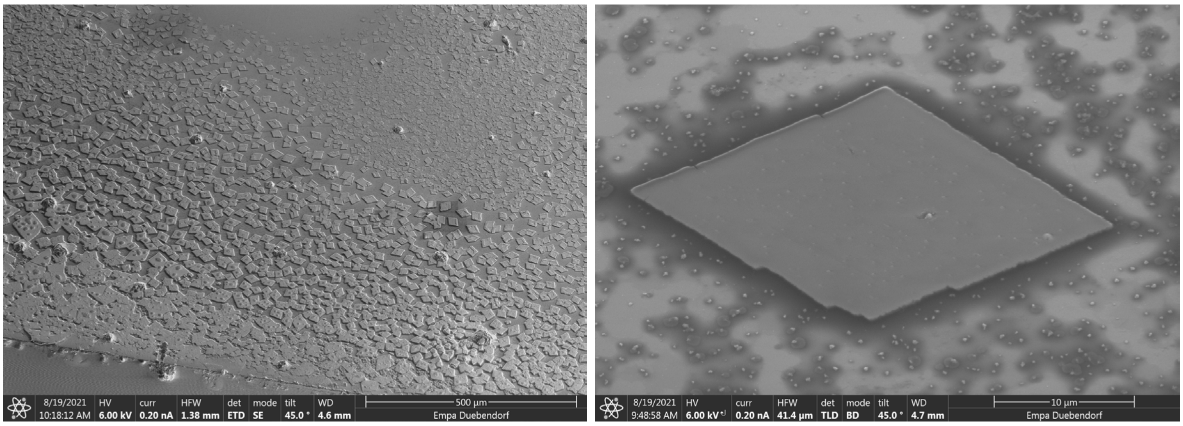

Figure S2 | Additional SEM images. *Left:* SEM image of densely packed SLs, demonstrating the predominance of the monoclinic shape discussed in the main text; towards the substrate edge (lower-left corner), the increased density of SLs leads to SL merging. *Right:* Higher-resolution SEM image of a single SL demonstrating uniform SL thickness.

Additional STEM images

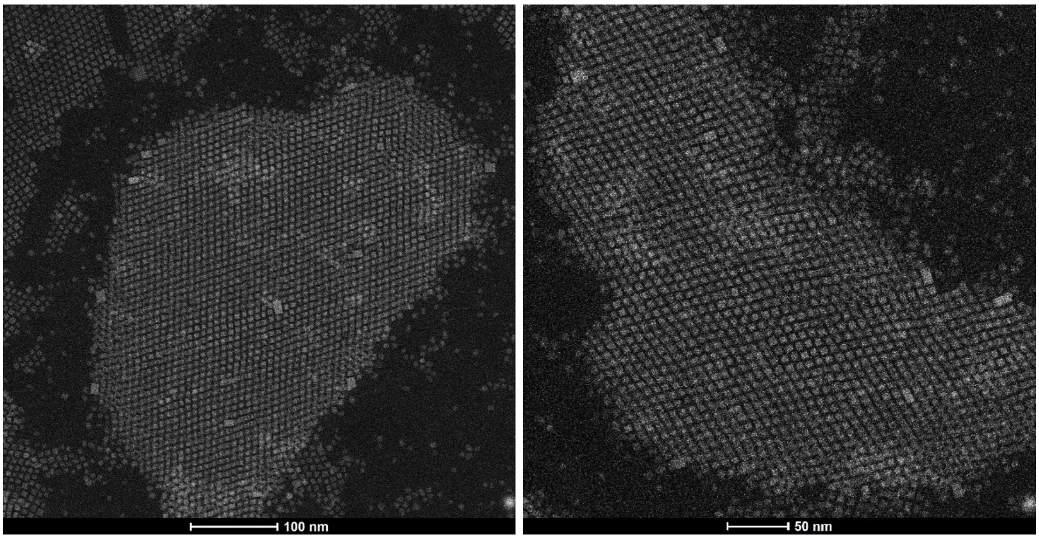

Figure S3 | STEM images of small SLs with a thickness of a few QD monolayers only.

HAADF-STEM tilting experiments

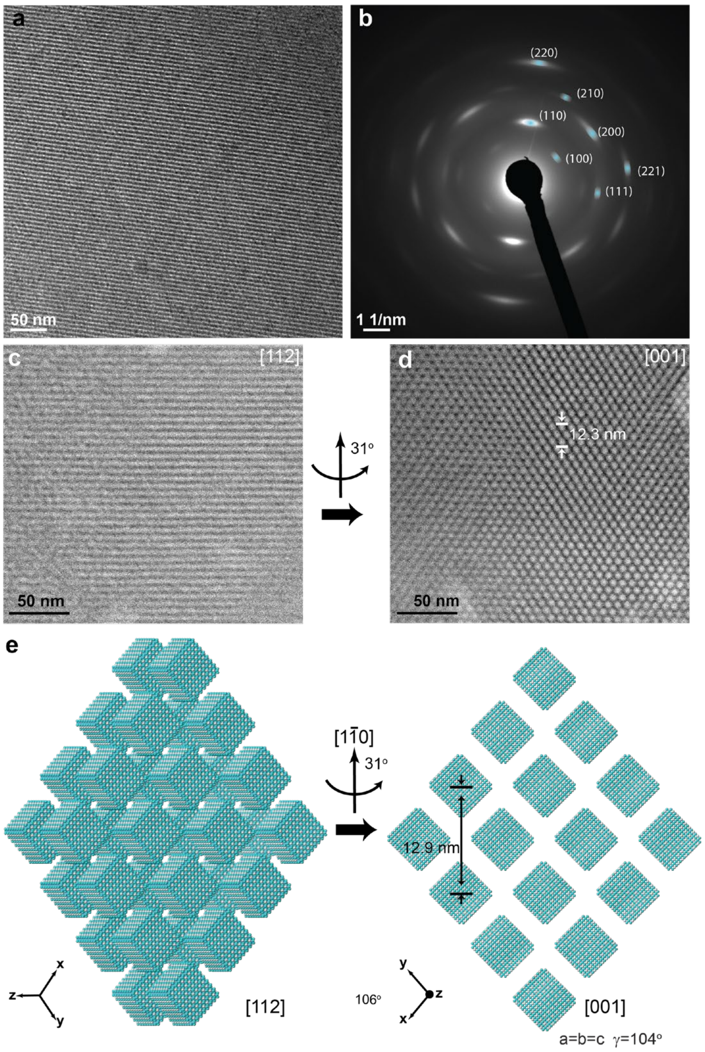

Figure S4. | HAADF-STEM tilting experiments. (a) TEM image and (b) corresponding wide-angle selected area electron diffraction of SL domain with [112] crystallographic orientation comprising 5 nm $CsPbBr_3$ QDs. (c, d) HAADF-STEM tilting experiment of a $[112]_{SL}$ domain around the $[1\text{-}10]_{SL}$ axis, before (c) and after (d) a 31° tilt. (e) Suggested QD SL model at the projections corresponding to the tilt experiments in (c) and (d), respectively. The indicated "diagonal" distance between QDs in the SL orientation [1-10] in our model (12.9 nm) is in good agreement with the experimentally determined distance (12.3 nm) in (d).

Image recognition methodology for optical microscopy images

We start by converting an input image from an optical microscope into a gray-scale image, with a single channel containing the light intensity. After a pre-processing step to sharpen the image utilizing the open-source computer vision library OpenCV,[9] edge detection is performed via thresholding. A fixed-level threshold ultimately converts the image into a binary one. Here, we would like to note that while the currently applied threshold level was arbitrary, and thus subject to some user bias, future implementation could reduce statistical bias via a suitable descriptor, *e.g.* based on particle concentration. Figure S6 shows a comparison of the image before and after processing.

Original image | **Image after sharpening** | **Image after thresholding**

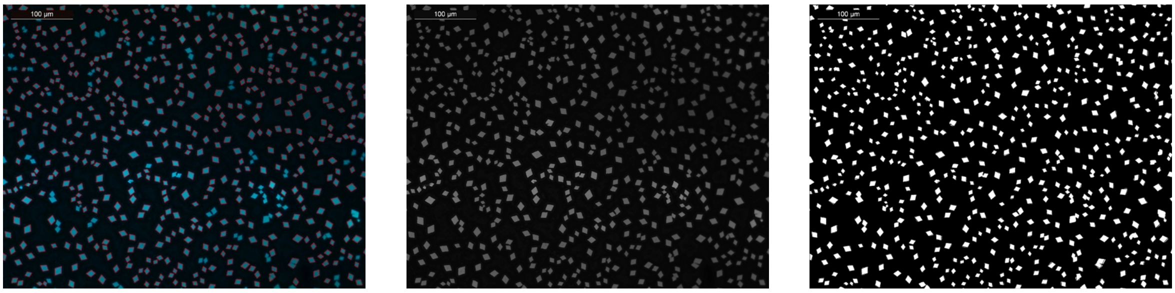

Figure S6 | Sequential steps in the image recognition algorithm.

Next, we utilize the OpenCV library[9] to perform a two-step contour search on the binary image, as an essential tool for shape analysis and object detection and recognition. After an initial contour search, obvious failures are rejected based on considerations of the expected minimum and maximum size of a detected object. Each remaining detected object is approximated by a polygonal curve consisting of four segments. Using basic linear algebra, we obtain the norms of the detected vectors and the enclosed angles. As a final result, we plot the histogram of all angles (four per detected object), as shown in the inset of Figure 2a in the main text, also reproduced here as Figure S7. Clearly, the detected angles suggest that QD SLs exhibit a non-orthogonal shape, with a characteristic obtuse angle of about 104°.

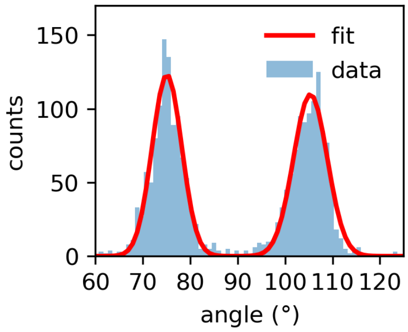

Figure S7 | Histogram of all angles enclosed by the detected shapes using our image-recognition algorithm. Identical to the lower inset in Figure 2a of the main text.

Atomistic model inferred from DSE fitting of the WAXTS data

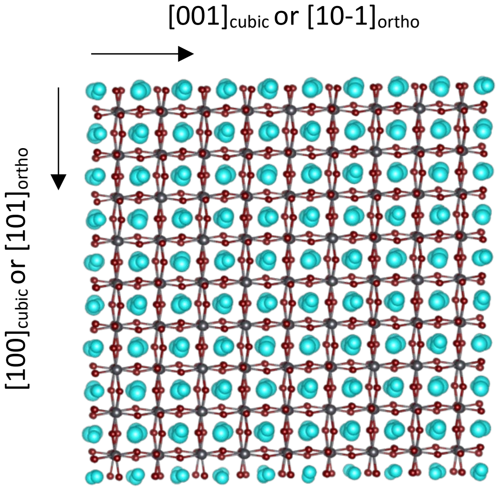

Figure S8. Atomistic model of a 3D $CsPbBr_3$ QD (viewed down [010] in both cubic and orthorhombic *Pnma* settings), corresponding to the best "average" DSE model. Such QD shows a cuboidal morphology (exposing either six {100} cubic facets, or, equivalently, four {101} and two {010} orthorhombic ones), a slip plane according to the ½<101>(010) rule (in orthorhombic notation) and stoichiometry Cs:Pb:Br = 1.0:1.0:3.2.

Average QD size derived from DSE fits of the Porod small-angle region and WAXTS data

The simultaneous DSE fitting of the Porod small-angle region and WAXTS data displayed in Figure 1d of the main text yields an oblate shape with average edge lengths of 4.5 nm, 5.2 nm, and 5.2 nm, respectively, and size dispersions (*i.e.* standard deviations of the bivariate log-normal distribution) of 17.2%, 7.8%, and 7.8%, respectively, corroborating the SAXS-derived values displayed in Figure 1b of the main text. The edge lengths derived and the standard deviations from the DSE fitting are mass-based average values.

Additional SAXS pattern

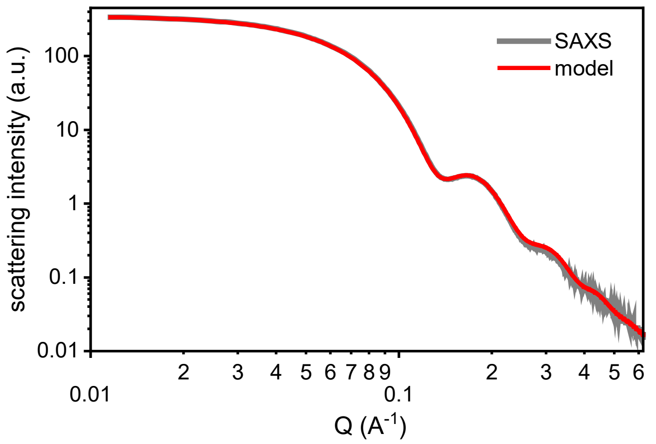

Figure S9 | SAXS data of a different synthesis batch than reported in the main text. SAXS data (grey line) and fit via the cuboidal model described in the main text (red line), yielding QD edge lengths of a = 4.5±0.1 nm, b = 5.4±0.2 nm, c = 5.5±0.2 nm, and a polydispersity of 9.5±3%.

GISAXS – the importance of accounting for the form factor

In a small X-Ray scattering experiment of particles with both positional and orientational order, the detected scattering intensity $\vec{I}(\vec{Q})$ can be written as

$$\vec{I}(\vec{Q}) = \vec{F}(\vec{Q}) \cdot \vec{S}(\vec{Q})$$

where $\vec{Q}$ denotes the scattering vector, $\vec{F}(\vec{Q})$ denotes the particle form-factor and $\vec{S}(\vec{Q})$ denotes the structure factor - note that all variables are given as vectors in 3D reciprocal space. In the following, we address the relevant aspects causing the observed modulation of Bragg-peak intensities, as shown in Figure 2i of the main text.

The scattering vector $\vec{Q}$ is determined by the experimental geometry, so the X-Ray incidence angle as well as the scattering angle with respect to the sample orientation. The particle form-factor $\vec{F}(\vec{Q})$ is governed by the QD shape. As an example, the solution SAXS pattern of the diluted QDs presented in Figure 1b of the main text shows the spherically averaged form factor $F(Q) = \langle\vec{F}(\vec{Q})\rangle$. The structure factor $\vec{S}(\vec{Q})$ is governed by the relative arrangements of individual scattering entities (atoms for GIWAXS and QDs for GISAXS), which can be defined, in the simplest cases, by the lattice periodicity only, or, more often, by the relative location of the scatterers within a unit cell. Hence, $\vec{S}(\vec{q})$ contains direction-dependent

information on the SL symmetry (crystal system and space group), periodicity (lattice parameter(s)), and "quality" (size, mosaicity, etc.).

As it is evident from the equation above, if a scattering intensity is measured at a specific $\vec{\boldsymbol{Q}}$, both form factor and structure factor need to be non-zero. Vice versa, if the either form factor or structure factor is zero, then also the scattering intensity will be supressed.

An evident example of this phenomenon is found in the horizontal cuts of the GISAXS patterns shown in Figure 2j of the main text. Here, the dashed lines indicate Q positions where one would expect SL Bragg peaks thanks to the in-plane order, yet some are experimentally unobserved. By superimposing the theoretical form factor of the QDs (calculated, for simplicity, assuming monodispersity with QD rhombic edges normal to the SL axis c_{SL}) it becomes evident that the experimentally missing Bragg reflections fall directly within the minima of the QD form factor. Specifically, this explains why Bragg peaks at about 0.12 Å^{-1} and 0.20 Å^{-1} are highly suppressed, while the Bragg peak in between, at 0.15 Å^{-1}, is strongly enhanced (as it is located near a strong form-factor maximum). A prominent example of this effect has been discussed by M.R. Jones et al.[10]

GISAXS – discarded alternative structural models

As counterproof of the proposed **C-centered** orthorhombic cell of the SL (which, as discussed, is nothing else than a simple textbook transformation of the primitive monoclinic 8.2 x 8.2 x 8.2 nm, γ = 104° lattice), we also attempted to index the obtained GISAXS patterns using an analogous **primitive** orthogonal lattice, maintaining cell edges lengths and forcing γ to 90°. A comparison between the spot indexing obtained by **primitive** monoclinic and **primitive** orthogonal lattices (which, *inter alia*, possess very different molar volumes) is shown in Figure S10. As neither the real nor the reciprocal lattice coincide (or are at least commensurate), in Figure S10c a few intense spots remain unindexed (eliminating the overlay of theoretical peak positions, SL Bragg reflections are more clearly visible). *Ab initio* indexing of the **all** observed spots (performed by GIDInd, doi.org/10.1107/S1600576721006609), using a joint Q_{xy} and Q_z dataset (with Q_z values taken from specular diffraction measurements), found a plausible *c-unique* **primitive** monoclinic lattice solution, nicely matching crystal morphology and leading to an overall orthorhombic lattice symmetry by simple axis transformation. Understandably, after transformation, the **C-centered** orthorhombic cell enables recovery of *hkl* triplets for all observed spots.

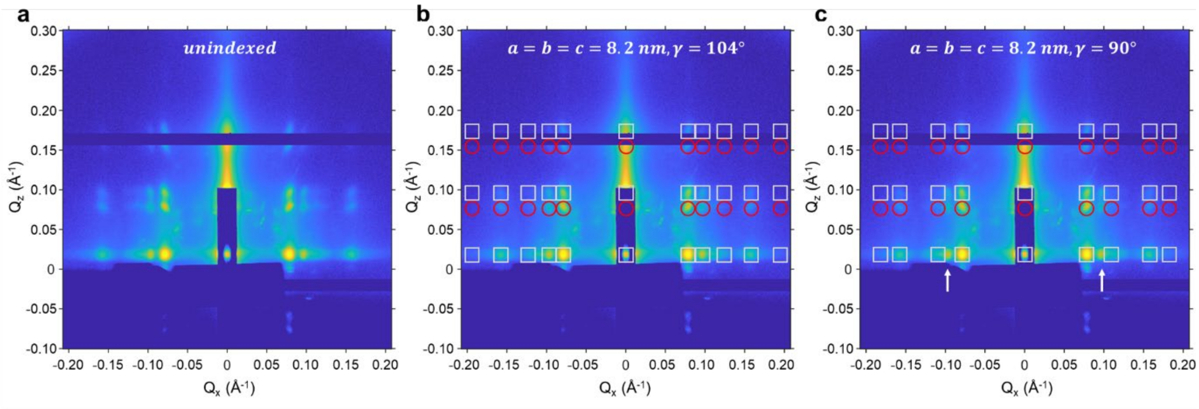

Figure S10 | Indexing of GISAXS images using alternative SL symmetries. (a) Raw (unindexed) GISAXS scattering image, clearly showing strong diffraction peaks. (b) The same scattering image shown in (a) with overlaid indexing for a monoclinic SL (8.2 x 8.2 x 8.2 nm, γ = 104°, as proposed by *ab-initio* indexing); grey and red markers indicate superimposition of two distinct (just Q_z translated) diffraction patterns resulting from instrumental effects and originating from QD interactions with "scattered" and "transmitted" beams, respectively. (c) The same scattering image shown in (a) and (b) with overlaid indexing corresponding to a **primitive** orthorhombic SL 8.2 x 8.2 x 8.2 nm, imposing γ = 90°. Note that the diffraction family indicated by the white arrow cannot be indexed in the context of a primitive orthogonal 8.2 x 8.2 x 8.2 nm SL.

GISAXS of additional QD SLs

To provide evidence of reproducibility of the formation of the proposed C-centered orthorhombic superlattice, we used the equivalent primitive monoclinic setting to interpret independently measured GISAXS images, acquired on a second sample more than one year after the initial measurements (those presented in Figure 2 of the main text and in Figure S10 above). In this late experiment, the sample-detector distance was adjusted to ca. 1.8 m, whereas images were taken at an incidence angle of 0.23° (aligned using the specular reflection).

As shown in Figure S11, also the new 2D GISAXS pattern could be indexed using a primitive monoclinic lattice. Here, the retrieved lattice parameters are a = b = 8.2 nm, c = 7.8 nm (with the c axis slightly reduced likely due to evaporation-induced compression effects, as observed in F. Krieg et al.[11]), and γ = 102°. This indexing further provides compelling evidence that the C-centered orthorhombic symmetry is maintained, as it only depends on the a = b relationship, and not on the c axis length or γ angle value. Overall, the agreement between these two independently obtained QDs, SLs, and GISAXS measurements evidences the outstanding repeatability of QD synthesis, SL growth, measurement stability, and lattice indexing.

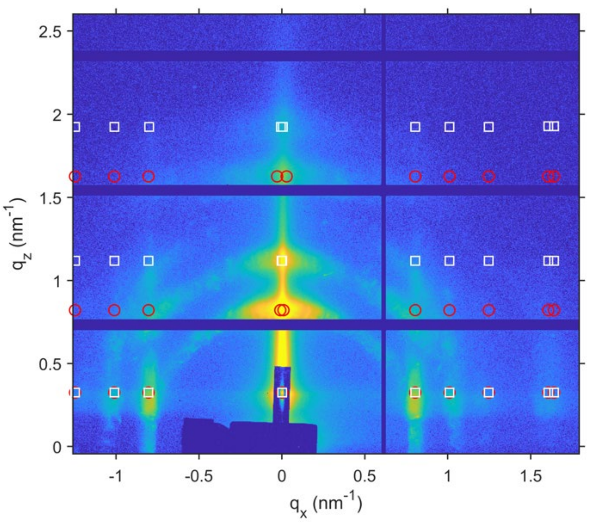

Figure S11 | GISAXS image of an additional QD SL. Also in this sample, a high long-range in-plane and out-of-plane order is observed. Indices correspond to the monoclinic symmetry with a = b = 8.2 nm, c = 7.8 nm, an in-plane angle of 102° and orthogonal out-of-plane angles; white and red markers indicate diffraction patterns resulting from the instrumental effects presented above.

Projected density of states via density functional theory

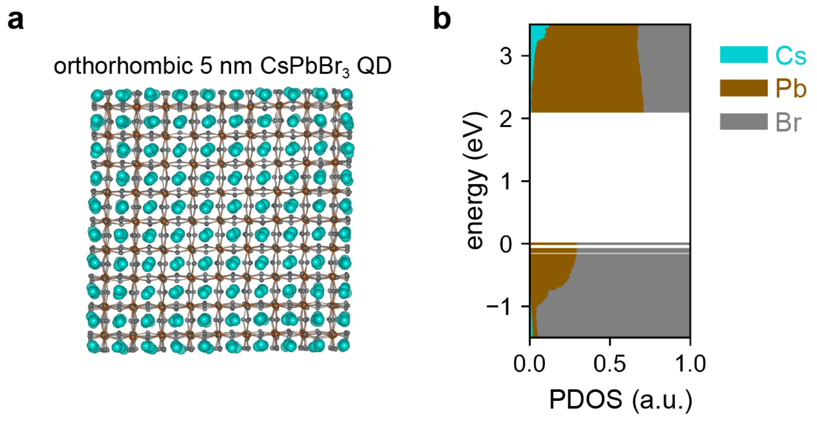

Figure S12 | Projected density of states of a 5 nm $CsPbBr_3$ QD. (a) Atomistic model of a 5 nm $CsPbBr_3$ QD, employed in the DSE fitting of the WAXTS data, after geometry relaxation at the DFT/PBE level of theory. The model is identical to the one shown in Figure 1e of the main text. (b) Associated projected density of states (PDOS) for the $CsPbBr_3$ QD, calculated at the DFT/PBE level of theory, with contributions by Cs, Pb, and Br shown in cyan, brown, and grey, respectively; the energy is referenced to the valence band edge.

Here, we assess the electronic structure of strongly confined 5 nm $CsPbBr_3$ from a computational point of view. Figure S12a reproduces the atomistic model utilized in the DSE fitting of the WAXTS data, see Figure 1e of the main text. Figure S12b shows the associated projected density of states (PDOS) calculated at the DFT/PBE level of theory using the CP2K software package. The clean bandgap, void of trap states, is consistent the high PL quantum yield achieved in the experiment. The band edge states predominantly derive from Pb and Br atomic orbitals, as typical also for larger $CsPbBr_3$ nanocrystals and bulk.

Control of the QD packing via the ligand length

In order to explore the effect of the capping ligand length on the self-assembly of 5 nm $CsPbBr_3$ QDs, we replace the DDAB ligands (didodecyldimethylammonium bromide, $(C_{12}H_{25})_2(CH_3)_2NBr$, with sidechains comprising 12 carbons) with other quaternary ammonium salts of either shorter or longer hydrocarbon sidechains, specifically:

- shorter ligand: dimethyldioctylammonium bromide, *i.e.* $(C_8H_{17})_2(CH_3)_2NBr$, with sidechains comprising 8 carbons, see Figure S13a,
- longer ligand: dimethyldioctadecyl ammonium bromide, *i.e.* $(C_{18}H_{37})_2(CH_3)_2NBr$, with sidechains comprising 18 carbons, see Figure S13b.

Two-dimensional SLs were prepared based on the controlled evaporation of the colloidal solutions on TEM grids.

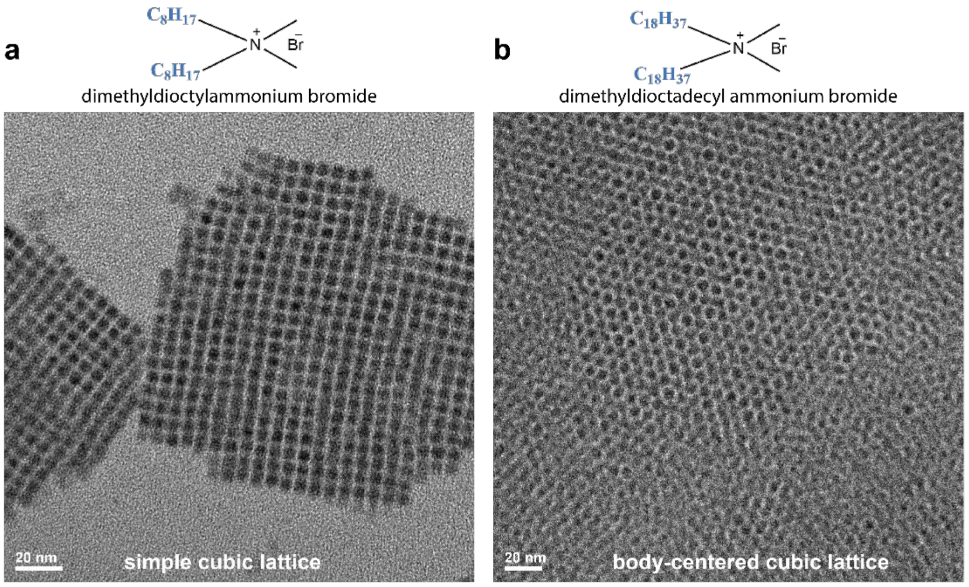

Figure S13 | Control of the QD packing via the ligand length. TEM images of two-dimensional superlattices comprising 5 nm $CsPbBr_3$ QDs capped with (a) short $(C_8H_{17})_2(CH_3)_2NBr$ and (b) long $(C_{18}H_{37})_2(CH_3)_2NBr$ ligands, respectively.

The softness of the 5 nm $CsPbBr_3$ nanocubes can be expressed as L/R (with L being the length of the capping ligands, and R the radius of the inorganic core) and increases for the considered quaternary ammonium bromides as $(C_8H_{17})_2(CH_3)_2NBr$ < $(C_{12}H_{25})_2(CH_3)_2NBr$ < $(C_{18}H_{37})_2(CH_3)_2NBr$.

In the case of the long $(C_{18}H_{37})_2(CH_3)_2NBr$ ligands, the larger softness value L/R leads to a "rounding" of the cuboidal shape of the 5 nm $CsPbBr_3$ NCs. Self-assembly of such QDs results in the body-centered cubic type SL shown in Figure S13b, a SL often observed for nearly spherical NCs with a significant soft character (see *e.g. J. Phys. Chem. Lett. 2015, 6, 13, 2406–2412* and *Nature Materials 2016, 15, 775–781*). In contrast, the installation of shorter $(C_8H_{17})_2(CH_3)_2NBr$ capping molecules on the surface of 5 nm $CsPbBr_3$ NCs yields simple cubic SLs (see Figure S13a), due to the decreased softness L/R and, concomitantly, well-defined edges and corners of the cuboidal building blocks. Such observations support our hypothesis that the large volume fraction of soft DDAB ligands on the surface of our 5 nm $CsPbBr_3$ nanocubes is key to explaining their atypical self-assembly into SLs of rhombic shape. More generally, engineering the softness of NCs via their ligand fraction is one way to direct the SL self-assembly into various lattice structures.

Thermal broadening of single-QD PL spectra

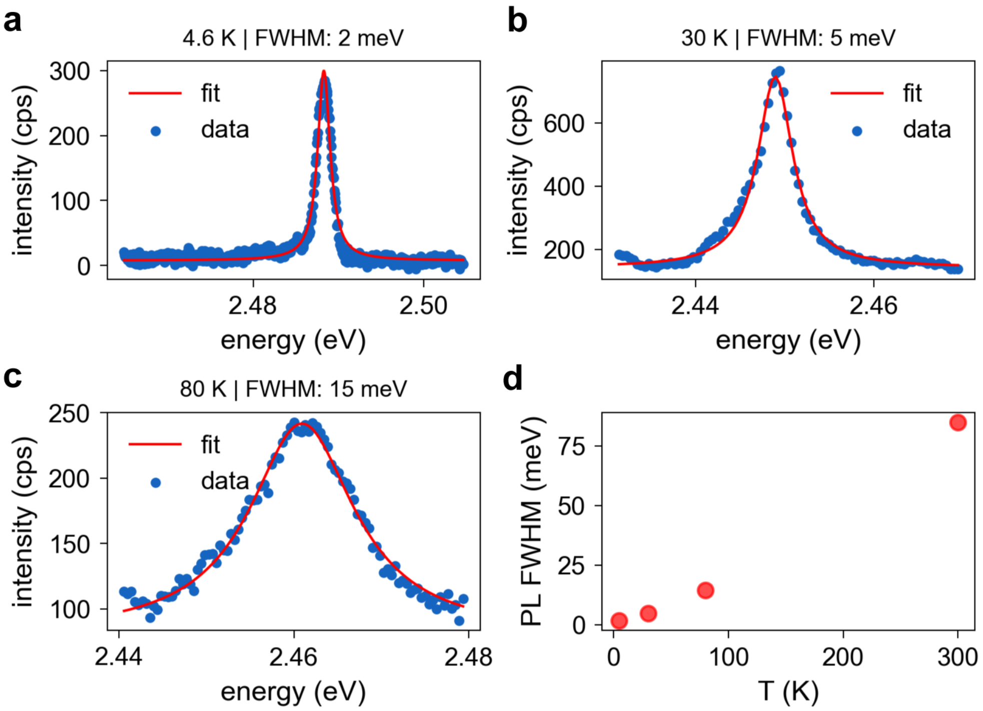

Figure S14 | Thermal broadening in single 5 nm $CsPbBr_3$ QDs. PL spectra (blue markers) and associated Lorentzian fits (red lines) in representative single 5 nm $CsPbBr_3$ QDs, at 4.6 K (a), 30 K (b), and 80 K (c), respectively. (d) The temperature-dependent PL broadening (fwhm), including the room-temperature data point (85 meV) from Figure 3a of the main text.

In a recent study, see *Nature Communications 2022, 13, 2587,* we have experimentally and computationally addressed the magnitude and origin of size-dependent homogeneous (thermal) broadening in $CsPbBr_3$ QDs, highlighting the pivotal role of the dynamic QD surface in phonon-induced emission broadening. In line with the findings of this prior work, we find here particularly pronounced PL broadening in small 5 nm $CsPbBr_3$ QDs with large surface-to-volume ratios. As shown in Figure S14, the PL linewidth (full width at half maximum, fwhm, obtained via Lorentzian fitting) of single 5 nm QDs increases strongly from 2 meV at 4 K to 5 meV at 30 K, 15 meV at 80 K, and 85 meV at 300 K, respectively. In compiling the temperature dependence shown in Figure S14d, spanning from 4 K up to 300 K, we took care to select representative QDs in terms of their broadening. Following a single QD across the entire temperature range was not possible, due to the still limited optical stability of such small QDs.

Temperature-dependent blinking characteristics in single 5 nm $CsPbBr_3$ QDs

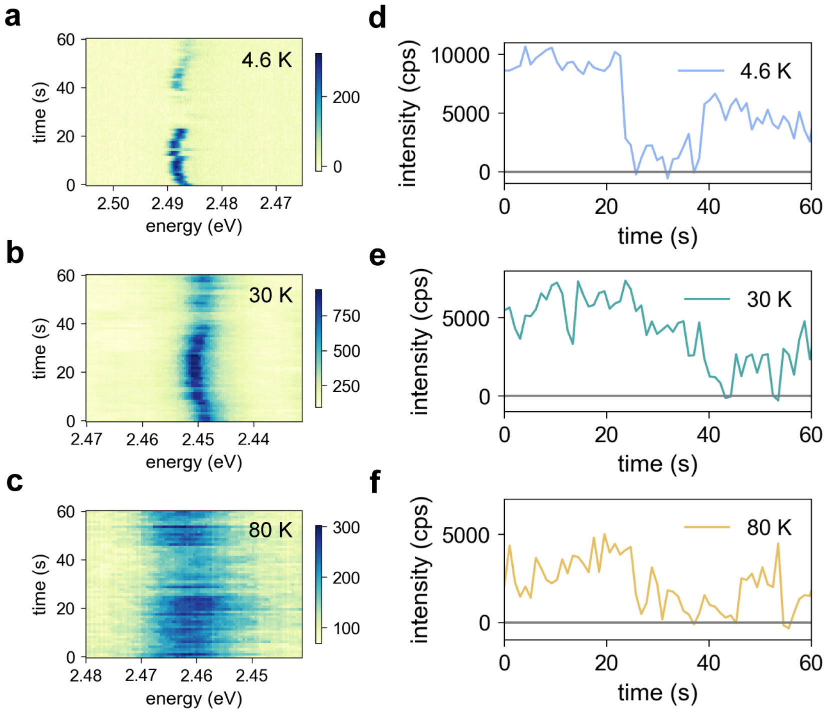

Figure S15 | Temperature-dependent blinking characteristics in single 5 nm $CsPbBr_3$ QDs. PL spectra were recorded at 4.6 K (a), 30 K (b), and 80 K (c), respectively, over the course of 60 s, with a 1-s integration time. Associated blinking traces at 4.6 K (d), 30 K (e), and 80 K (f), respectively, have been obtained from (a)-(c) via spectral integration across the PL peak, after background subtraction.

Commonly, cooling down QDs can suppress PL intensity blinking, as *e.g.* reported for II-VI QDs (see *e.g. Phys. Rev. B 2001, 63 205316*) and larger $CsPbBr_3$ QDs (see *e.g. ACS Nano 2016, 10, 2, 2485–2490*). However, PL blinking in our 5 nm QDs still persists at cryogenic temperatures, as clearly observed when acquiring consecutive PL spectra. Suppressing PL

blinking at cryogenic temperature still requires further work on the surface passivation and optimization of sample preparation routines. Indeed, strong dilution factors, needed to prepare sparse QD films, are possibly the cause of a non-optimal ligand surface coverage in single-QD samples which could be responsible for the observed blinking phenomenon.

REFERENCES

(1) Glatter, O. Determination of particle-size distribution functions from small-angle scattering data by means of the indirect transformation method. *J. Appl. Cryst.* **1980**, 13, 7–11.

(2) Pedersen, J. S. Analysis of small-angle scattering data from colloids and polymer solutions: modeling and least-squares fitting. *Adv. Colloid Interface Sci.* **1997**, 70, 171–210.

(3) Debye, P. Zerstreuung von Röntgenstrahlen. *Annalen der Physik* **1915**, *351* (6), 809-823.

(4) Bertolotti, F.; Moscheni, D.; Guagliardi, A.; Masciocchi, N. When crystals go nano – the role of advanced X-ray total scattering methods in nanotechnology. *Eur. J. Inorg. Chem.* **2018**, *2018* (34), 3789-3803.

(5) Cervellino, A.; Frison, R.; Bertolotti, F.; Guagliardi, A. DEBUSSY 2.0: the new release of a Debye user system for nanocrystalline and/or disordered materials. *J. Appl. Crystallogr.* **2015**, *48* (6), 2026-2032.

(6) Bertolotti, F.; Nedelcu, G.; Vivani, A.; Cervellino, A.; Masciocchi, N.; Guagliardi, A.; Kovalenko, M. V. Crystal structure, morphology, and surface termination of cyan-emissive, six-monolayers-thick $CsPbBr_3$ nanoplatelets from X-ray total scattering. *ACS Nano* **2019**, *13* (12), 14294-14307.

(7) Bertolotti, F.; Protesescu, L.; Kovalenko, M. V.; Yakunin, S.; Cervellino, A.; Billinge, S. J. L.; Terban, M. W.; Pedersen, J. S.; Masciocchi, N.; Guagliardi, A. Coherent nanotwins and dynamic disorder in cesium lead halide perovskite nanocrystals. *ACS Nano* **2017**, *11* (4), 3819-3831.

(8) Cervellino, A.; Giannini, C.; Guagliardi, A. On the efficient evaluation of Fourier patterns for nanoparticles and clusters. *J. Comput. Chem.* **2006**, *27* (9), 995-1008.

(9) *OpenCV: Open Source Computer Vision Library*; 2022. https://github.com/opencv/opencv

(10) Jones, M. R.; Macfarlane, R. J.; Lee, B.; Zhang, J.; Young, K. L.; Senesi, A. J.; Mirkin, C. A. DNA-nanoparticle superlattices formed from anisotropic building blocks. *Nat. Mater.* **2010**, *9* (11), 913-917.

(11) Krieg, F.; Sercel, P. C.; Burian, M.; Andrusiv, H.; Bodnarchuk, M. I.; Stöferle, T.; Mahrt, R. F.; Naumenko, D.; Amenitsch, H.; Rainò, G.; et al. Monodisperse long-chain sulfobetaine-capped $CsPbBr_3$ nanocrystals and their superfluorescent assemblies. *ACS Cent. Sci.* **2021**, *7* (1), 135-144.

(12) Kühne, T. D., et al. CP2K: An electronic structure and molecular dynamics software package - Quickstep: Efficient and accurate electronic structure calculations. *J. Chem. Phys.* **2020**, 152, 194103.
